# Valorization of Rice By‐Products Through Conventional and Emerging Extraction Technologies: Trends, Challenges, and Applications

**DOI:** 10.1111/1541-4337.70511

**Published:** 2026-05-18

**Authors:** Shabnam Haghighat Khajavi, Aidin Taromsari, Kiana Moradi, Maria Concetta Tenuta, Giovanna Ferrentino

**Affiliations:** ^1^ Faculty of Agricultural, Environmental and Food Sciences Free University of Bozen‐Bolzano Bolzano Italy; ^2^ Department of Food Science and Technology, Science and Research Branch Islamic Azad University Tehran Iran

**Keywords:** bioactive compounds, by‐products valorization, circular economy, functional properties, hybrid techniques, innovative extraction technologies

## Abstract

Rice and by‐products, such as rice bran (RB), defatted RB (DRB), rice husk (RH), rice straw (RS), and broken rice, represent abundant yet underutilized resources within the rice value chain. These fractions are nutritionally dense, containing proteins, lipids, dietary fibers, phenolics, and other bioactives that can be transformed into high‐value food, nutraceutical, cosmetic, and bio‐based products. However, their industrial use has long been limited by rapid enzymatic deterioration, structural heterogeneity, and reliance on solvent‐intensive legacy methods. This review consolidates current knowledge on the composition of rice by‐products and systematically compares extraction technologies across three categories: conventional (e.g., maceration, Soxhlet, and acid/alkali digestion), green (e.g., pressurized liquid and subcritical water extraction, supercritical CO_2_, microwave‐ and ultrasound‐assisted processes, pulsed electric fields, ohmic heating, cold plasma, and enzyme‐assisted extraction), and hybrid approaches. Special attention is given to cascade and integrated strategies that combine complementary mechanisms to enhance selectivity, preserve thermolabile compounds, reduce solvent and energy consumption, and improve scalability. Beyond technical aspects, the review maps how extracted fractions translate into application‐ready ingredients such as lipid concentrates, protein hydrolysates, dietary fibers, prebiotic oligosaccharides, pigments, and mineral complexes. It also highlights decision criteria, compound polarity, heat sensitivity, and processing scale, which can guide technology selection in practice. Finally, future perspectives emphasize the importance of standardization, techno‐economic, life‐cycle assessments, and regulatory harmonization to accelerate the deployment of rice by‐product valorization within sustainable and circular bioeconomy frameworks.

## 1 Introduction

Rice is one of the most important staple foods worldwide, feeding more than half of the global population. Its industrial processing generates large quantities of by‐products, including rice bran (RB), defatted RB (DRB), rice husk (RH), rice straw (RS), and broken rice, the latter being a fraction generated during milling that differs from whole rice only in kernel size and not in nutritional composition (Aluthge et al. 2023; Das et al. 2025; Zhou et al. 2025). These by‐products represent a valuable yet underutilized biomass resource, as they are rich in proteins, lipids, dietary fibers, phenolic compounds, vitamins, and minerals, making them promising raw materials for applications in the food, nutraceutical, cosmetic, and bio‐based sectors (Fărcaș et al. 2022; Punia et al. 2021; Sapwarobol et al. 2021).

Despite this compositional richness, the effective valorization of rice by‐products remains challenging. Their industrial exploitation is often limited by rapid enzymatic deterioration, particularly in lipid‐rich fractions such as RB, as well as by matrix heterogeneity, structural recalcitrance, and the limited selectivity of conventional extraction approaches (Borrelli and Ficco 2025; Chemat et al. 2020). Traditional techniques such as maceration, Soxhlet extraction, and acid/alkali digestion are still widely used, but they often require long extraction times, large solvent volumes, high energy input, and may compromise the stability of thermolabile bioactive compounds (Gil‐Martín et al. 2022; Kumar et al. 2023). In addition to their environmental burden, these methods may promote chemical and structural degradation of sensitive constituents, such as lipid oxidation, carotenoid isomerization, vitamin loss, and phenolic transformation, thereby reducing both functional quality and application potential.

To address these limitations, increasing attention has been directed toward green and intensified extraction technologies capable of improving extraction efficiency while reducing environmental impact. These include pressurized liquid extraction (PLE), subcritical water extraction (SWE), supercritical CO_2_ extraction (SFE), microwave‐assisted extraction (MAE), ultrasound‐assisted extraction (UAE), pulsed electric fields (PEFs), ohmic heating, cold plasma (CP), and enzyme‐assisted extraction (EAE) (Buljeta et al. 2023; Khalid et al. 2024; Modupalli et al. 2024; Xi et al. 2024). Such approaches can enhance mass transfer, reduce solvent and energy consumption, improve selectivity, and better preserve thermolabile compounds compared with conventional methods. Moreover, hybrid and cascade extraction strategies, which combine complementary mechanisms or sequential recovery steps, are emerging as particularly promising tools for the integral valorization of complex biomass streams (Carpentieri et al. 2021; Tufail et al. 2024).

At the same time, the valorization of rice by‐products should not be viewed solely from the perspective of extraction yield. In recent years, research in functional foods and bio‐based ingredients has increasingly emphasized the importance of compound stability, bioaccessibility, bioavailability, and matrix interactions, alongside compositional recovery. Bioactive constituents such as polyphenols, carotenoids, phytosterols, soluble fibers, bioactive peptides, and structural polysaccharides are of particular interest due to their potential roles in the prevention of chronic diseases and in the development of value‐added food and nonfood products. Therefore, the selection of an extraction strategy should be guided not only by efficiency but also by the physicochemical nature of the target compounds, matrix complexity, thermal sensitivity, and intended end‐use application.

Although several review articles have addressed individual technologies or specific groups of bioactive compounds, a comprehensive and integrative overview focused specifically on rice processing by‐products is still lacking (Colla et al. 2025; Peanparkdee and Iwamoto 2019). In particular, there is a need for a review that systematically compares conventional, green, hybrid, and cascade extraction approaches while also linking these technologies to the composition, functionality, and potential applications of the recovered fractions.

Therefore, the aim of this review is to critically synthesize the current knowledge on the valorization of rice processing by‐products through extraction technologies, with emphasis on (i) the chemical composition and functional potential of the main rice‐derived side streams; (ii) the principles, advantages, and limitations of conventional, green, and hybrid extraction techniques; (iii) the relationship between matrix characteristics, compound properties, and extraction strategy selection; and (iv) the application potential of the recovered fractions in food and related sectors. In addition, this review highlights current research gaps and future priorities, including process standardization, techno‐economic feasibility, life‐cycle assessment (LCA), and regulatory harmonization. The literature survey was conducted using PubMed, ScienceDirect, and Web of Science, with keywords including *rice by‐products*, *conventional and green extraction of rice by‐products*, *bioactive compounds in rice by‐products*, and *applications of rice by‐products*. A total of approximately 285 records were initially identified, and after screening for relevance and quality, 171 articles were included in this review. Only peer‐reviewed articles published in English from 2000 onward were considered. Studies were included on the basis of their relevance to composition, extraction performance, technological advantages and limitations, and food‐related applications. Articles were excluded if they were not focused on rice by‐products, lacked experimental or review‐based scientific data, or were not aligned with the scope of this study.

## 2 Rice By‑Products Composition

Rice by‐products, such as bran, husk, and broken kernels, are increasingly valued as sources of bioactive compounds, nutrients, and functional ingredients. Rice by‐products derived from pigmented (e.g., red, black, and purple) and nonpigmented rice (white and brown) exhibit significant differences in composition, extraction behavior, and functional applications. Pigmented rice by‐products are particularly rich in phenolic compounds, flavonoids, and anthocyanins, which contribute to their strong antioxidant and anti‐inflammatory potential, whereas nonpigmented RB is generally characterized by higher levels of lipophilic bioactives, such as γ‐oryzanol, tocopherols, tocotrienols, and essential fatty acids (Deng et al. 2013). More broadly, cereal by‐products from wheat, barley, oats, maize, and rice are increasingly recognized as important reservoirs of phytochemicals, including phenolic acids, flavonoids, carotenoids, alkylresorcinols, sterols, and dietary fiber‐associated antioxidants, although their abundance and distribution vary according to grain type, anatomical fraction, genotype, pigmentation, and processing history. Within this wider cereal context, rice by‐products are particularly noteworthy due to their combination of lipid‐soluble antioxidants, phenolic constituents, and nutritionally valuable fractions, which make them especially attractive for sustainable valorization and functional ingredient development (Fărcaș et al. 2022).

### 2.1 Rice Bran

**FIGURE 1:**
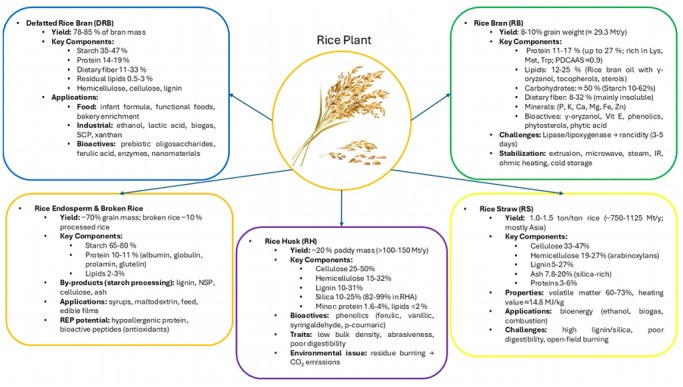
Rice by‐products and their compositional landscapes.

RB is the outer fraction obtained during the polishing of brown rice to white rice, typically accounting for 8%–10% of the paddy grain weight, with global production estimated at approximately 63 million tons per year (Aluthge et al. 2023; Zhuang et al. 2019). Reported yields vary from 5% to 12% (w/w), depending on cultivar, degree of milling (DOM), and processing method (Fărcaș et al. 2022; Modupalli et al. 2024; Punia et al. 2021; Rodríguez‐Restrepo et al. 2020; Sapwarobol et al. 2021; Soares et al. 2018; Spaggiari et al. 2021; Yılmaz Tuncel 2023). RB retains about 60% of the nutrients of the whole grain, far exceeding polished rice (Begum et al. 2015; Guazzotti et al. 2023; Spaggiari et al. 2021). From a compositional perspective, RB is characterized by protein contents typically ranging from 11% to 17%, with some reports up to 27% (Figure 1). These proteins are rich in lysine, methionine, and tryptophan, with PDCAAS values around 0.90, underscoring their nutritional quality (Colla et al. 2025; KC et al. 2024; Liu et al. 2019; Manzoor et al. 2023; Mathias et al. 2023; Roy et al. 2025; N. Wang et al. 2023; Yılmaz Tuncel 2023; Zaky et al. 2022). Lipids comprise 12%–25% of RB, primarily in the form of RB oil (RBO), which is enriched in γ‐oryzanol, tocopherols, tocotrienols, and sterols (Baixinho et al. 2025; Balegh et al. 2025; Colombo et al. 2024; Tufail et al. 2024). Carbohydrates account for roughly half of RB; digestible carbohydrates represent around 18%, whereas starch content ranges widely from 10% to 62%, reflecting genotype and DOM variations (Colla et al. 2025; Dang and Vasanthan 2019; Lavanya et al. 2019; Rashid et al. 2022; Sapwarobol et al. 2021). Dietary fiber is reported between 8% and 32% (commonly 12%–20%), largely insoluble (Alexandri et al. 2020; Fărcaș et al. 2022; Zhou et al. 2025). Ash levels of 4.9%–17% provide essential minerals, including phosphorus, potassium, calcium, magnesium, iron, and zinc (Fraterrigo Garofalo et al. 2021; Illankoon et al. 2023).

RB also contains a wide array of bioactive compounds, including γ‐oryzanol (152–912 mg/100 g dry bran; 4%–6% of crude oil), vitamin E homologues (tocopherols and tocotrienols), phenolic acids (ferulic, caffeic, gallic, and syringic), phytosterols, squalene, phytic acid (IP6), lipoic acid, and coenzyme precursors (Balegh et al. 2025; Chen et al. 2019; Colombo et al. 2024; Lavanya et al. 2019; Peanparkdee and Iwamoto 2019; Zaky et al. 2022; Zullaikah et al. 2019). These compositional features vary with genotype, environment, and milling degree, with higher DOM typically reducing lipid and fiber content due to greater endosperm contamination (Fraterrigo Garofalo et al. 2021; Manzoor et al. 2023; Spaggiari et al. 2021; Yılmaz Tuncel 2023).

A major limitation of RB is its susceptibility to rancidity within 3–5 days of milling due to lipase and lipoxygenase activity being effective on lipids (triacylglycerols) to produce free fatty acids (FFAs), thus resulting in a soapy taste and aroma. Effective stabilization strategies, including extrusion, microwave heating, steam treatment, infrared radiation (IR), ohmic heating, and cold storage, are therefore essential for extending shelf life and maintaining functionality (Borrelli and Ficco 2025; Chen et al. 2019; Liu et al. 2019; Rashid et al. 2023; Spaggiari et al. 2021; Yılmaz Tuncel 2023).

RB has been one of the most studied by‐products for nutritional and functional valorization. In the years, it has been treated with conventional extraction techniques, including organic solvents and thermal treatments, for the recovery of phenol‐ and lipid‐rich fractions (Rodríguez‐Restrepo et al. 2020). In the last years, the use of green extraction techniques, such as UAE, MAE, EAE, and SWE, allows the preservation of thermolabile bioactive compounds and increases the extraction efficiency of soluble fibers and bound phenols (Fărcaș et al. 2022; Aluthge et al. 2025). These approaches also reduce the solvent use and energy consumption. The extracts obtained with these techniques presented higher antioxidant activity and provided more selective yields compared to conventional methods, making the recovered compounds suitable for food and nutraceutical applications (Galanakis 2022).

### 2.2 Defatted Rice Bran

DRB, also referred to as de‐oiled RB (DORB), is the solid residue remaining after RBO extraction and represents approximately 78%–85% of the original bran mass (Alexandri et al. 2020). Its proximate composition typically includes starch (35%–47.5%), protein (14%–19%), dietary fiber (11%–33%; both soluble and insoluble), residual lipids (0.5%–3%), structural polysaccharides (hemicellulose, cellulose, and lignin), and significant amounts of essential minerals (Dang and Vasanthan 2019; Modupalli et al. 2024; N. Wang et al. 2023).

Proteins in DRB exhibit a favorable amino acid balance, high digestibility, and hypoallergenic potential, making them promising ingredients for infant formulas, clinical nutrition, and functional foods. In bakery systems, DRB incorporation enhances fiber content, antioxidant capacity, and shelf life while maintaining acceptable sensory properties when properly formulated (Aluthge et al. 2023; Roy et al. 2025; Spaggiari et al. 2021; Zaky et al. 2022). Beyond food applications, DRB serves as a versatile substrate for industrial bioprocesses. It supports microbial fermentations for the production of bioethanol, lactic acid, xanthan gum, biohydrogen, biogas, and single‐cell proteins (Colombo et al. 2024; Dang and Vasanthan 2019; Illankoon et al. 2023; Kyu et al. 2019; Nidhishree et al. 2024). Additionally, DRB provides a valuable source of prebiotic oligosaccharides, ferulic acid, enzymes, and starch‐based nanomaterials (Colla et al. 2025; Guazzotti et al. 2023; Sapwarobol et al. 2021).

### 2.3 Rice Endosperm

The RE constitutes about 70% of the grain mass, whereas broken rice accounts for approximately 10% of processed volume, mainly utilized in starch industries (Fărcaș et al. 2022; Roy et al. 2025; Sapwarobol et al. 2021; Totaro et al. 2018). The endosperm is predominantly starchy, with a composition of 65%–80% starch, 10%–11% protein, 2%–3% lipids, and small fractions of cellulose, lignin, ash, and dietary fiber (Mondal et al. 2021; Totaro et al. 2018).

Rice endosperm protein (REP) is composed of albumin, globulin, prolamin, and glutelin fractions, the latter representing 60%–80% of total protein. REP is relatively rich in lysine compared to other cereal proteins, but its solubility is limited due to extensive disulfide cross‐linking (Roy et al. 2025). By‐products derived from starch processing show a composition of 64.8 wt% starch (with approximately 10% α‐amylase‐resistant fraction), 12.7 wt% lignin, 7.5 wt% non‐cellulosic polysaccharides, 3.5 wt% cellulose, 1.6 wt% ash, and trace extractives (<0.1 wt%) (Totaro et al. 2018).

Broken rice is widely valorized into glucose and fructose syrups, maltodextrin, nutraceuticals, feed, extrusion products, and edible films (Sapwarobol et al. 2021; Totaro et al. 2018). REP also serves as a hypoallergenic protein source, with digestibility and amino acid profiles comparable to soy, whey, and casein. Hydrolyzed REP generates bioactive peptides with antioxidant potential, expanding its applications in functional foods and nutraceuticals (Babini et al. 2020; Fabian et al. 2011; Jafarzadeh et al. 2025; KC et al. 2024; Liu et al. 2019; N. Wang et al. 2023).

### 2.4 Rice Husk

RH forms around 20% of paddy mass, yielding >100–150 Mt/year globally; reports include 199 Mt in 2018 and 102 Mt projected for 2023/24 (Nzereogu et al. 2023; Singh and Patel 2022; Yılmaz Tuncel 2023; Yuan et al. 2024).

Compositionally, RH contains cellulose 25%–50%, hemicellulose 15%–32% (with prominent arabinoxylans), lignin 10%–31%, and silica 10%–25% (dry weight), which concentrates to 82%–99% in RH ash (RHA). Minor components include proteins 1.6%–4.1%, lipids <1%–2%, moisture 5%–16%, and minerals (Ca, Mg, Mn, Na, Fe, K) (Brites et al. 2024; Colombo et al. 2024; Illankoon et al. 2023; Ishida et al. 2025; Jaichakan et al. 2021; Kamboj et al. 2024; Lee et al. 2025; Nzereogu et al. 2023; Requena et al. 2019; Samsalee et al. 2023; Steven et al. 2021; Yeboah et al. 2022; W. Zhang et al. 2023). RH also carries phenolics, ferulic, vanillic, syringaldehyde, and *p*‐coumaric acids, supporting antioxidant potential (Butsat et al. 2009; Colombo et al. 2024; Illankoon et al. 2023; Kaur and Ubeyitogullari 2023). Key physical traits are low bulk density (around 96–160 kg/m^3^), abrasiveness, and poor digestibility (Nzereogu et al. 2023; Steven et al. 2021). Open‐field residue burning remains a major concern, contributing approximately 54.6 Mt CO_2_‐eq in 2017 (Alexandri et al. 2020; Kaur and Ubeyitogullari 2023; Singh and Patel 2022).

### 2.5 Rice Straw

RS, consisting of stems, leaves, and spikelets, is produced at 1.0–1.5 ton per ton of rice, with ranges from 0.41 to 3.96 t depending on cultivar and agronomic practices. RS can reach 50% of total crop dry biomass, corresponding to a global output of approximately 750–1125 Mt/year, with estimates of 1058 Mt in 2018, mostly concentrated in Asia (China and India; 289 and 232 Mt, respectively) (Dafiqurrohman et al. 2022; Illankoon et al. 2023; Islam et al. 2022; Kamboj et al. 2024; Ly et al. 2024; Peanparkdee and Iwamoto 2019; Singh and Patel 2022; Van Hung et al. 2020; W. Zhang et al. 2023). The compositional profile of RS includes cellulose (33%–47%), hemicellulose (19%–27%; arabinoxylans around 14.2%), lignin (5%–27%), and silica‐rich ash (7.8%–20.3%). Proteins occur at 3%–6%, lipids at 1.6%, and extractives such as pectin, waxes, and chlorophylls are also present (Dafiqurrohman et al. 2022; Illankoon et al. 2023; Islam et al. 2022; Jaichakan et al. 2021; Kamboj et al. 2024; Van Hung et al. 2020; W. Zhang et al. 2023). Thermochemical indices show volatile matter of 60.6%–73.4%, fixed carbon 11.1%–16.8%, and a lower heating value of approximately 14.8 MJ/kg, underscoring its potential for bioenergy applications (Illankoon et al. 2023; Islam et al. 2022). Constraints to RS utilization include widespread open‐field burning, which contributed 54.6 Mt CO_2_‐eq emissions in 2017 (Alexandri et al. 2020; Kaur and Ubeyitogullari 2023; Singh and Patel 2022), as well as intrinsic limitations such as high lignin and silica content, low digestibility, poor protein and mineral availability, abrasiveness, and low bulk density (Illankoon et al. 2023; Kamboj et al. 2024; Ly et al. 2024; Van Hung et al. 2020). Nevertheless, recent studies have highlighted that RS should be reconsidered as a valuable lignocellulosic resource rather than a waste material, with significant potential for sustainable valorization. Biorefinery approaches also suggested the possibility of converting it into biofuels, biochar, adsorbent materials for pollutant removal, and functional biomaterials for industrial applications, thus supporting the transition toward a circular bioeconomy (Martínez‐Guillén et al. 2025).

## 3 Conventional Extraction Methods

Extraction plays a crucial role in biomass valorization by facilitating the recovery of proteins, lipids, phenolics, and other high‐value compounds. Historically, traditional extraction techniques, such as maceration, infusion, decoction, boiling and hot water extraction, percolation and Soxhlet, acid and alkali extraction, thermal rendering, and mechanical aids, have formed the basis for processing RB and other plant by‐products, serving as benchmarks against which modern green technologies are assessed (Arellano et al. 2022; Gil‐Martín et al. 2022; Usman et al. 2023). Although these methods are straightforward with a simple industrial scalability and widely accessible with relatively low operational costs, they require extensive solvent use, leading often to the degradation of thermolabile compounds. Nonetheless, their contribution to establishing biomass extraction processes remains essential (Chemat et al. 2020; Colombo et al. 2024). The choice of the solvent is dependent on the polarity of the bioactive compounds to extract. As an example, hexane is the primary solvent used for the extraction of RBO due to its low cost, low boiling point, and high solubility. However, residual hexane in the oil matrix can be toxic to humans. Over the years, researchers have explored alternative solvents to address this issue. Isopropyl alcohol and cyclohexane have also been tested. Nevertheless, the quality of the extracted oil has been quantified as lower than that obtained using hexane. Despite these alternatives, hexane remains the mainstream solvent in industrial RBO production due to its economic advantages (Ribas et al. 2023; Wang et al. 2022). Zullaikah et al. (2019) reported that hexane in Soxhlet extraction from RB yielded 17%–18% of oil characterized with high γ‐oryzanol content. Benito‐Román et al. (2019) similarly obtained high oil yields using this approach. Moreover, the conventional methods present long extraction time and large volume of solvents and may increase environmental pollution (Lee et al. 2013). In the past decades, an increasing attention has been posed to green extraction techniques that are able to shorten the extraction times and reduce the use of organic solvent consumption (Sarfarazi et al. 2020).

## 4 Green Extraction Methods

**FIGURE 2:**
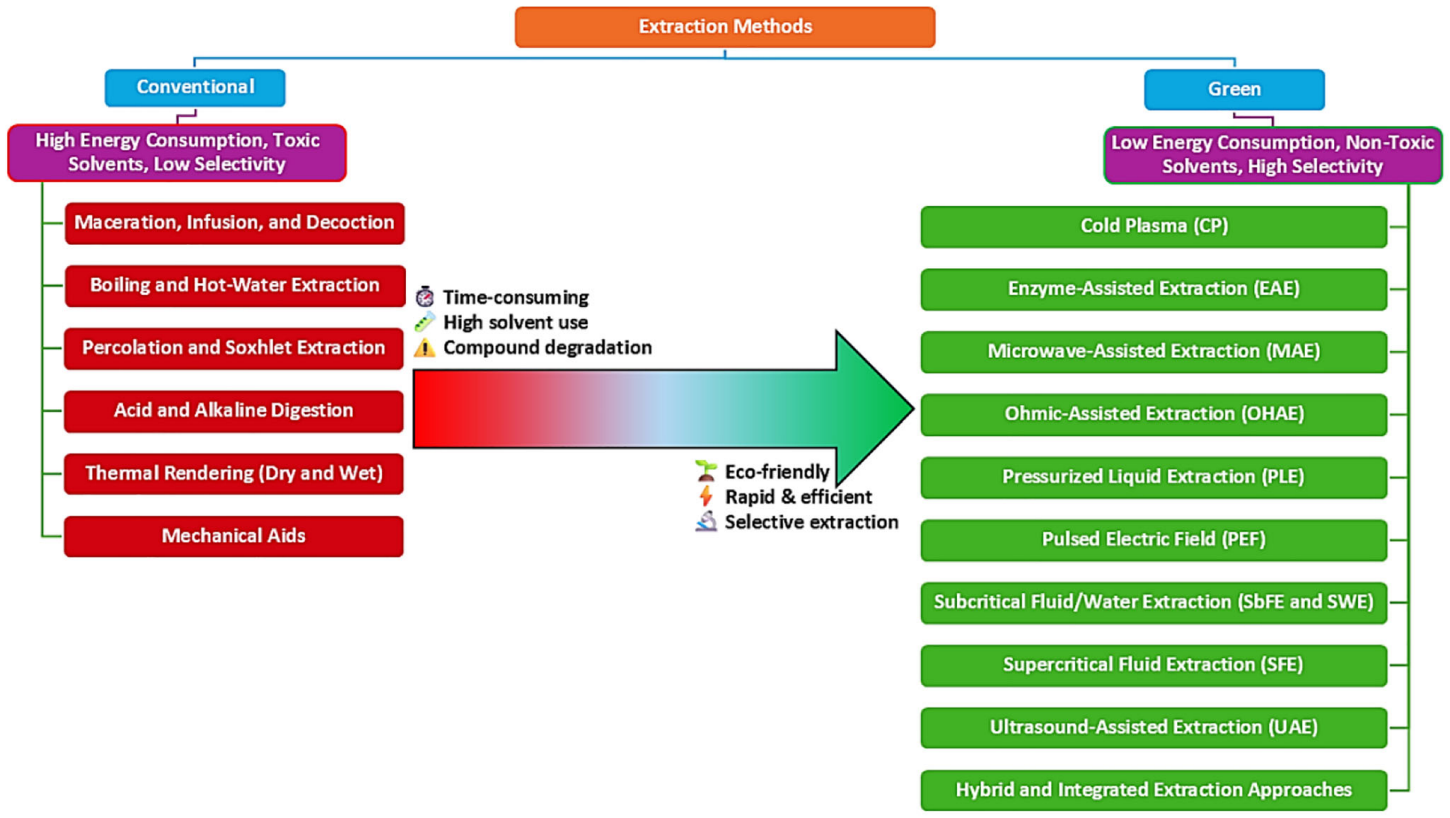
Comparison of conventional and green extraction methods for biomass valorization.

Compared to conventional techniques, green extraction methods offer significant advantages in terms of nutritional preservation, process efficiency, and environmental sustainability. One of the most important advantages highlighted across the studies has been the enhanced bioactivity of the recovered compounds and nutrients. As an example, Kovačević et al. (2018) demonstrate that innovative extraction techniques improve the recovery and stability of phenolic compounds compared to a conventional extraction with solvent. The study published by Sarfarazi et al. (2020) also reports enhanced bioactivity of plant‐based extracts from *Crocus sativus*, obtained through advanced processing techniques. In addition, emerging technologies may improve the extraction yields, shorten processing times, and reduce energy consumption. For example, UAE and MAE significantly reduce processing time from hours to minutes while increasing extraction efficiency. A recent study conducted by Pankaj et al. (2025) emphasizes that these technologies not only improve productivity and inactivate microorganism, preserving the structural and nutritional integrity of food products, but also reduce operational costs. The emerging technologies applied for the valorization of rice by‐products include CP, ASE, MAE, UAE, SFE and EAE. Collectively, these approaches, along with hybrid strategies, provide a faster, cleaner, and more selective means of recovering bioactives (Figure 2) (Chemat et al. 2020; Modupalli et al. 2024; Usman et al. 2023). Overall, three key parameters can guide green extraction selection: compound polarity, matrix recalcitrance, and thermal sensitivity. Nonpolar or lipophilic compounds are generally more amenable to extraction with supercritical CO_2_, whereas polar and semipolar compounds often require the use of co‐solvents or alternative green extraction approaches, such as UAE or PLE. Similarly, highly recalcitrant matrices, characterized by rigid cell walls or strong compound–matrix interactions, may require pretreatment strategies or intensified techniques to enhance mass transfer and compound release. In the case of thermolabile bioactives, mild operating conditions become essential to preserve compound integrity and bioactivity, making low‐temperature or short‐residence‐time technologies more appropriate. Therefore, extraction process design should be approached through a matrix–compound compatibility perspective, enabling a more rational and application‐driven selection of sustainable extraction technologies. In the present review, to improve cross‐method interpretation, Table 1 comparatively summarizes the main extraction technologies applied to rice by‐products, including their operating principles, target compounds, representative process conditions, performance trends, advantages, limitations, and relative cost/scalability.

**TABLE 1:** Comparative performance of conventional, green, and hybrid extraction technologies for rice by‐product valorization.

Extraction technology	Main mechanism	Typical target compounds	Typical operating/Optimal conditions^a^	Reported performance/Yield trend	Main advantages	Main limitations	Relative cost/Scalability
Maceration/Solvent extraction	Diffusion‐driven solid–liquid extraction	Phenolics, lipids, pigments, semipolar compounds	Ambient to moderate temperature; long extraction time (hours–days); ethanol, methanol, hexane, acetone, water depending on polarity	Low‐to‐moderate yield; often lower than intensified methods	Simple, low equipment requirement, easy implementation, suitable as benchmark	Long extraction time, high solvent use, low selectivity, possible degradation/oxidation, poor sustainability	Low CAPEX, easy scale‐up, but low process efficiency
Soxhlet extraction	Continuous hot solvent reflux	Rice bran oil, lipophilic compounds, some phenolics	Solvent boiling temperature; several hours; commonly *n*‐hexane, petroleum ether, ethanol	Moderate‐to‐high oil recovery; often used as reference method	High extraction completeness, reproducible, standardized	Long time, high solvent consumption, thermal stress, not ideal for thermolabile compounds	Low–moderate cost, scalable but environmentally burdensome
Acid/Alkali extraction	Solubilization/Hydrolysis of matrix components	Proteins, polysaccharides, fibers, silica, hemicellulose	pH‐adjusted extraction; moderate‐to‐high temperature; chemical hydrolysis	Moderate‐to‐high recovery depending on matrix	Effective for structural biopolymers and proteins, widely used	Chemical consumption, wastewater generation, possible structural damage, reduced bioactivity	Low reagent cost, scalable, but environmental burden high
Enzyme‐assisted extraction (EAE)	Enzymatic hydrolysis of cell walls/macromolecular complexes	Oils, proteins, peptides, phenolics, soluble fibers, oligosaccharides	Usually 35–60°C, controlled pH, hours; cellulase, xylanase, protease, pectinase, amylase or enzyme cocktails	Moderate to high; often markedly improved over aqueous extraction; highly matrix‐dependent	Mild conditions, high selectivity, improved functionality, food‐grade, reduced solvent use	Long process time, enzyme cost, emulsion formation, scale‐up complexity, enzyme sensitivity	Moderate OPEX, scalable with optimization, industrially promising
Ultrasound‐assisted extraction (UAE)	Acoustic cavitation, cell disruption, enhanced mass transfer	Phenolics, flavonoids, anthocyanins, oils, proteins, polysaccharides	Commonly 20–40 kHz, 5–30 min, mild–moderate temperatures, ethanol/water mixtures frequently used	High extraction efficiency in short time; often superior‐to‐conventional methods	Fast, reduced solvent use, mild thermal load, simple integration as pretreatment or stand‐alone	Nonuniform cavitation, possible localized heating/radical formation, scale‐up challenges	Moderate cost, good pilot/industrial potential
Microwave‐assisted extraction (MAE)	Rapid volumetric dielectric heating, cell rupture, accelerated diffusion	Oils, phenolics, tocopherols, γ‐oryzanol, anthocyanins, pigments	Commonly 500–1000 W, short treatment (seconds–minutes), ethanol/water or polar solvents	High yield and fast kinetics; often better than Soxhlet/maceration	Very short extraction time, low solvent demand, efficient energy transfer	Risk of overheating, nonuniform heating, degradation of heat‐sensitive compounds if not controlled	Moderate cost, good scalability with process control
Ohmic‐assisted extraction (OHAE)	Internal resistive heating + electroporation	Oils, proteins, anthocyanins, phenolics, pectin, fibers	Electrical field typically 50–225 V/cm, short treatment, conductive/moist matrices required	Moderate to high; often improves stabilization and extractability	Rapid and uniform heating, reduced hot spots, good retention of bioactives, useful for stabilization	Electrode corrosion, dependence on matrix conductivity, equipment cost, process standardization needed	Moderate–high cost, promising but less mature industrially
Pulsed electric field (PEF)	Electroporation of cell membranes	Phenolics, proteins, pigments, intracellular metabolites	Usually 0.1–80 kV/cm, microsecond–millisecond pulses, very short treatment	Moderate‐to‐high enhancement when used as pretreatment; often boosts downstream extraction	Nonthermal, preserves thermolabile compounds, low solvent requirement, short treatment	High equipment cost, limited effect in dense/low‐conductivity matrices, optimization complexity	Moderate–high CAPEX, strong industrial promise mainly as pretreatment
Cold plasma (CP)	Reactive species generation, surface etching, membrane disruption	Phenolics, proteins, lipids, antioxidant compounds	Usually atmospheric or low‐pressure plasma; treatment in seconds–minutes; gas composition and power dependent	Moderate‐to‐high enhancement; especially useful as pretreatment/stabilization aid	Nonthermal, short treatment, low solvent need, improved accessibility and stabilization	Reactor complexity, limited penetration depth, oxidative damage risk, scale‐up still challenging	High equipment complexity, emerging technology
Pressurized liquid extraction (PLE/ASE)	High‐pressure solvent extraction at elevated temperature	Phenolics, oils, anthocyanins, semipolar and nonpolar compounds	Typically 50–250°C, 4–20 MPa, 5–20 min, ethanol/water commonly used	High to very high; often near‐exhaustive extraction with low solvent volume	Fast, efficient, programmable, reproducible, reduced solvent use, good for food‐grade solvents	High equipment cost, possible thermal degradation, co‐extraction of undesired compounds	High CAPEX, strong industrial relevance
Subcritical water extraction (SWE)	Water under subcritical conditions with reduced polarity	Phenolics, arabinoxylans, proteins, oligosaccharides, sugars, semipolar compounds	Typically 100–250/374°C, pressurized liquid water, short–moderate extraction time	High for polar/semipolar compounds; can simultaneously hydrolyze matrix	Solvent‐free (water only), green, selective tunability, suitable for biorefinery integration	Thermal degradation risk, furfural/HMF formation, corrosion, energy demand	High equipment cost, scalable but optimization‐critical
Supercritical fluid extraction (SFE, mainly SC‐CO_2_)	Solvation with supercritical fluids; density‐tunable extraction	Rice bran oil, γ‐oryzanol, tocopherols, tocotrienols, sterols, lipophilic compounds	Commonly 30–40 MPa, 40–60°C; ethanol often used as co‐solvent for polar compounds	High for lipophilic compounds; highly selective, high‐quality extracts	Solvent‐free final product, mild temperature, selective, excellent for oils and sensitive lipophiles	High capital cost, lower efficiency for polar compounds without co‐solvent, process complexity	High CAPEX, industrially relevant for high‐value fractions
Subcritical fluid extraction (SbFE; e.g., propane/butane systems)	Pressurized liquid/gas extraction below critical point	Oils, lipophilic bioactives, sterols, antioxidants	Moderate pressure, lower temperature than Soxhlet; solvent‐dependent	High oil extraction efficiency with good quality	Efficient for lipids, lower thermal stress, reduced solvent residues compared with hexane	Solvent safety concerns, equipment requirements, limited suitability for polar compounds	Moderate–high cost, niche industrial potential
Hybrid approaches (e.g., UAE + EAE, UAE + MAE, PEF + SFE, SWE + CP, MAE + UAE)	Combination of complementary mechanisms	Multi‐fraction recovery: oils, proteins, phenolics, fibers, sugars, pigments	Technology‐specific; often sequential or pretreatment‐assisted optimization required	Very high/synergistic in many cases; often better than single methods	Higher yield, better selectivity, shorter time, lower solvent demand, suitable for cascade valorization	Process complexity, high optimization burden, scale‐up and standardization still limited	Moderate‐to‐high cost, highly promising for integrated biorefineries

Abbreviations: ASE, accelerated solvent extraction; CAPEX, capital expenditure; OPEX, operational expenditure; SC‐CO_2_, supercritical carbon dioxide.

^a^Operating conditions are reported as representative ranges commonly described in the literature and may vary depending on rice fraction (rice bran, defatted rice bran, rice husk, rice straw, and broken rice), target compound, solvent system, and equipment configuration. “Reported performance/yield trend” is expressed qualitatively because direct numerical comparison across studies is often limited by differences in raw material, pretreatment, extraction basis, and analytical methodology.

### 4.1 Cold Plasma Extraction

CP has emerged as a promising nonthermal and environmentally friendly method for extracting bioactive compounds from agri‐food by‐products, particularly those derived from rice processing streams (Khalid et al. 2024; Mehta et al. 2022; Nidhishree et al. 2024; Xi et al. 2024). Treatment times are generally short (minutes), with medium–high equipment costs due to specialized reactors. CP provides high yield of phenolics, proteins, and lipids while preserving thermolabile compounds, thanks to its ambient processing temperature. In addition, it offers low energy consumption and reduced solvent use. It also enhances surface hydrophilicity and can improve matrix stability when combined with other innovative technologies, such as ultrasound (Khalid et al. 2024). CP consists of an ionized gas made up of free electrons, ions, radicals, and ultraviolet photons. Although it remains electrically neutral on a macroscopic scale, it is highly reactive at the molecular level (Misnal et al. 2022; Zhou et al. 2025). Under nonequilibrium conditions, the electron temperatures greatly exceed those of heavier particles, allowing the bulk system to remain close to ambient temperatures (approximately 300 K) and rarely exceeding 1000 K. This unique nonthermal characteristic prevents the degradation of thermolabile compounds while also offering advantages such as brief treatment times, low energy consumption (15–300 W), and the absence of toxic residues (Khalid et al. 2024; Xi et al. 2024). The mechanisms underlying CP‐mediated extraction entail both physical and chemical modifications of plant tissues. Reactive oxygen and nitrogen species (ROS/RNS), such as O_3_, •OH, H_2_O_2_, NO, and NO_2_, are generated by nonthermal plasmas in either gaseous or aqueous forms when primary plasma species (ions, electrons, radicals, and dissociated molecules) interact with a liquid phase. Charged particles and electric fields induce surface etching, pitting, and microcrack formation, increasing surface roughness and accessibility by reducing diffusion resistance and facilitating solvent penetration (Balegh et al. 2025; Mehta et al. 2022). The CP treatment reduces crystallinity, making the product less physically stable. This effect is attributed to the degradation of molecules in the extract matrix induced by energetic collisions between ROS and the molecules during CP extraction. These interactions cause structural modifications that increase the amorphous regions, thereby reducing the overall crystallinity (Yadav et al. 2021). Additionally, electroporation deforms cell membranes to create micropores, promoting the release of intracellular molecules, including phenolics, proteins, and oils (Modupalli et al. 2024; Murakonda and Dwivedi 2024). Murakonda and Dwivedi (2024) observed that the combination of CP with UAE initially disrupts the matrix structure, promoting the release and solubilization of interesting molecules. Subsequently, ultrasound treatment contributes to partial restoration of crystallinity, improving the structural stability of the matrix.

The applications of CP encompass a wide variety of biomolecules. Research has shown that this method enhances the extraction of phenolic acids (such as ferulic, chlorogenic, and *p*‐coumaric acids), proteins derived from oilseed cakes, and lipids like RBO (Khalid et al. 2024; Mehta et al. 2022; Talha et al. 2024). In the case of RB, CP not only preserves antioxidant capacity, digestibility, and anti‐inflammatory activity but also surpasses conventional extraction methods in both yield and bioactivity retention (Das et al. 2025; Nidhishree et al. 2024). Recent innovations in reactor design have aimed to enhance process homogeneity. For instance, multi‐pin atmospheric CP systems utilize dense pin arrays, such as 63 pins arranged in a 9 × 7 matrix, to achieve uniform discharges across thin layers of biomass (approximately 1 mm thick). The effectiveness of such treatments has been demonstrated in food by‐products. Mehta et al. (2022) applied atmospheric CP to DRB, achieving significant enhancements in total phenolic content and antioxidant capacity. Similarly, Zheng et al. (2023) showed that CP treatment of brown rice and brown rice powder effectively inactivated peroxidase activity while preserving bioactive compounds. Misnal et al. (2022) further observed a significant increase in γ‐aminobutyric acid (GABA) content following CP treatment of brown rice. More recently, Balegh et al. (2025) demonstrated that CP treatment of RB can improve both oil yield and bioactive compound retention. These findings highlight both the technological potential of improved CP reactor designs and their applicability in stabilizing rice‐based matrices while retaining nutritional functionality (Table 2). This approach results in extensive surface etching, increased phenolic and flavonoid content, and minimal thermal degradation (Murakonda and Dwivedi 2024).

**TABLE 2:** Green, hybrid, and conventional extraction methods.

Extraction technology	Extraction method	Valorized by‐product	Targeted compound	Processing conditions	References
Green	MAE (microwave‐assisted extraction)	Rice husk (RH)	Polyphenols	MAE with 80% ethanol–water (1:35 g/mL) at 500 W, 90°C for 5 min yielded 136,819.6 mAU s of polyphenols	Frosi et al. (2023)
	SWE (subcritical water extraction)	Defatted rice bran (DRB)	Fermentable sugars Platform chemicals	SWE of DRB with distilled water (50–100 g/g DRB) at 260°C and 10 MPa for 20 min produced 6.53 ± 0.61 g/100 g fermentable sugars and 9.55 ± 1.10 g/100 g platform chemicals	Moreira et al. (2023)
			Protein hydrolysate	SWE of DRB with distilled water (100 g/500 mL) produced protein hydrolysates with 30.31% ± 0.57% protein under conditions of 180°C, 20 MPa for 60 min	Wongthaweewatana et al. (2021)
		Rice husk (RH)	Glucose Xylose Mannose Arabinose Galactose Rhamnose 5‐HMF Furfural Acetic acid Formic acid	SWE of RH with distilled water (6 g/120 mL) at 150–350°C yielded glucose (0.24 mg/mL), mannose (0.06 mg/mL), xylose (0.12 mg/mL), arabinose (0.22 mg/mL), formic acid (0.45 mg/mL), acetic acid (2.05 mg/mL), furfural (2.50 mg/mL), 5‐HMF (0.80 mg/mL), and total reducing sugars (7.49 mg/mL)	Lee et al. (2025)
	SFE/SC‐CO_2_ (supercritical CO_2_ extraction)	Rice bran (RB)	γ‐Oryzanol Fatty acids	SC‐CO_2_ extraction of RB at 62°C and 500 bar for 3 h (15 g/min) achieved a yield of 17.3% ± 0.5%, containing 36.4 ± 3.5 mg/g γ‐oryzanol and 784.4 ± 18.4 mg/g fatty acids	Baixinho et al. (2025)
			Rice bran oil (RBO) γ‐Oryzanol	Supercritical CO_2_ extraction of rice bran at 40°C and 400 bar for 3 h (20 kg/h) yielded 37 g/kg rice bran oil (RBO) and 200 mg/kg γ‐oryzanol	Fraterrigo Garofalo et al. (2021)
			Rice bran oil (RBO)	SC‐CO_2_ extraction of rice bran at 40°C and 20 MPa for 120 min (8 g/min) achieved an oil yield of 17.47 ± 1.34 g/100 g RB	Moreira et al. (2023)
			Rice bran oil (RBO)	Supercritical CO_2_ extraction of rice bran at 40°C and 40 MPa (1.0 L/min) yielded 11.72 ± 0.48 to 15.60 ± 0.55 wt% rice bran oil, containing 13.37%–16.32% palmitic, 44.60%–52.56% oleic, and 29.90%–38.51% linoleic acids	Pinto et al. (2021)
	PEF (pulsed electric field)	Rice bran (RB)	Protein	PEF treatment of rice bran protein in distilled water (1:10 w/v) at pH 10 for 25 min with 2.3 kV/cm, 8 kV, and 250 pulses/min increased protein content by 20.71%–22.8%	Thongkong et al. (2023)
	Solvent‐based UAE (ultrasound‐assisted extraction)	Rice bran (RB)	Anthocyanins Phenolics Flavonoids	Solvent‐based ultrasound‐assisted extraction of pigmented RB using 60% aqueous methanol with 0.1% citric acid (1:20 g/mL) at 30°C and 37 kHz for 30 min yielded 3.15 ± 0.15 mg C3G/g DW anthocyanins, 16.13 ± 0.82 mg GAE/g DW phenolics, and 4.04 ± 0.11 mg QE/g DW flavonoids with an IC50 of 413.90 ± 47.46 µg/mL (DPPH)	Bunmusik et al. (2023)
	UAE (ultrasound‐assisted extraction)	Rice bran (RB)	Free phenolics (phenolic acids)	5 g RB + 65% EtOH, ultrasonic bath 48 kHz, 45 min, extract: DPPH IC50 (275.1 ± 13.79 µg/mL), ABTS IC50 (63.45 ± 6.21 µg/mL), highest free phenolics (19.73% ± 1.45% w/w)	Guerrini et al. (2020)
	PLE (pressurized liquid extraction)	Rice bran (RB)	Rice bran oil Co‐extracted phenolics β‐Sitosterol	Pressurized liquid extraction of rice bran with 99.5% ethanol under intermittent cycles at 95–109°C with static times of 7–13 min. The oil yield ranged from 24.83% to 27.22%, with the highest yield (27.22%) obtained at 107°C and 8 min	Echenique et al. (2024)
		Black rice bran (BRB)	Anthocyanin‐rich extract (notably cyanidin‐3‐O‐glucoside, C3G)	Continuous pressurized liquid extraction of black rice bran was carried out at 10 MPa for 8 min at 55°C, with a flow rate of 5 mL/min using an ethanol‐citric acid (0.1 mol/L) solution (50:50, v/v), recovered about 80% (observed 79.22% ± 2.68%) of total anthocyanin	Leonarski et al. (2023)
		Rice bran (RB)	Rice bran oil	Pressurized liquid extraction of rice bran was performed using 10 g of sample and 52 mL of ethanol (99.5%) at 107°C, for three cycles with 60% rinse volume and 8 min static time, yielded 130.42% rice bran oil	P. R. Ramos et al. (2024)
Biological (fermentation and enzyme)	EAE (enzymatic assisted extraction)	Rice bran (RB)	Physicochemical properties Fatty acid compositions Bioactive compounds Antioxidant activity Thermal behavior	Alcalase‐assisted enzymatic extraction of rice bran using 2 g/100 g bran of Alcalase 2.4 L in distilled water (1:7.5 w/v) at 57°C, pH 9 for 150 min produced extracts with 76.31% UFAs, 1004 mg/kg tocopherols/tocotrienols, EC50 of 5.52 mg/mL, and a peroxide value of 8.15 ± 0.44 mmol/kg	Xu et al. (2021)
			Soluble dietary fiber Soluble pentosan	EAE of washed RB using xylanase (10 mg/g (Enzeco, Ali, Bio‐cat) or 0.1 mL/g (Multifect 720) in distilled water (1:4 w/v) at 55°C for 5 h increased soluble content by >50%	Dang and Vasanthan (2019)
			Soluble dietary fiber	Defatted rice bran was enzymatically hydrolyzed with α‐amylase (75°C, 3.5 h, pH 6.0) and protease (60°C, 2.5 h, pH 8.0), followed by ethanol precipitation, centrifugation, drying, and milling to obtain RBSDF. This was incorporated into wheat flour dough at 125–205 µg/g	Zhang et al. (2024)
	SSF (solid‐state fermentation)	Rice endosperm (RE)	Phenolic content Flavonoid content Antioxidant activity Tyrosinase Elastase inhibition activities	SSF of BR with *Aspergillus niger* and *Rhizopus oligosporus* (5 × 10^6^ spores/g) in distilled water (35 mL/flask) at 32°C for 20 days yielded 4.77 mg GAE/g TPC, 0.90 mg QE/g TFC, 94.17% DPPH scavenging activity, 18.47 mM Fe(II)/g ferric‐reducing activity, 46% tyrosinase inhibition, and 51.9% elastase inhibition	Abd Razak et al. (2019)
			Total phenolics Total flavonoids Kojic acid Phenolic acids Tyrosinase inhibitory activity Elastase inhibitory activity Antioxidant activity	SSF of RB with *Aspergillus brasiliensis*, *Aspergillus awamori*, and *Aspergillus sojae* (1% v/w) in water (1:1 w/w) at 25°C for 8 days (*A. brasiliensis*) or 14 days (*A. awamor*i*, A. sojae*), followed by ethanol extraction (95%, 1:10 w/v), yielded TFC of 1.3 mg QE/g and TPC of 36.5 mg GAE/g (*A. sojae*), kojic acid (0.58 ± 0.011 mg/mL, *A. sojae*), sinapic acid (4.65 µg/mL, *A. awamori*), and ferulic acid (24.73 µg/mL, *A. awamori*), with IC50 of 1.55 mg/mL, 63.9% DPPH scavenging activity (*A. sojae*), and 85% elastase inhibition (*A. awamori*)	Ritthibut et al. (2021)
		Defatted rice bran (DRB)	Phenolic profiles Bio‐accessibility Antioxidant activity	SSF of DRB with Rhizopus oryzae (1 × 10^4^ spores/g) in distilled water (40 g DRB + 20 mL) at 30°C for 5 days, followed by extraction with 80% acetone, increased free phenolics by 99.4%, bound phenolics by 40%, and total phenolics by 71.6%, with ORAC activity enhanced by 141.8% and 45.3% (bound)	Chen et al. (2019)
		Rice protein by‐product	Peptides	Fermentation of rice protein by‐products with lactic acid bacteria strains LrC1122, Lp82, and LrPRRH at 37°C for 72 h produced 1.04–5.54 g/L protein hydrolysates enriched in bioactive peptides	Babini et al. (2020)
		Rice fiber (RF)	Cellulases	SSF of rice fiber using a starter mixture of rice fiber and compost (9:1 w/w) with wood chips as a bulking agent (1:1 v/v) followed by extraction in distilled water (1:1 w/v) at 25°C for 30 min achieved cellulase activity recovery of 139% ± 11%	Marín et al. (2019)
Hybrid	Supercritical CO_2_ (SCCO_2_) + ethanol (co‐solvent)	Rice bran (RB)	Parboiled rice bran oil	Supercritical CO_2_ extraction with ethanol (1:1) as a co‐solvent at 40°C, 200 bar for 250 min (45.94 g CO_2_/g bran) recovered 25.5 wt% parboiled rice bran oil	Trevisani Juchen et al. (2019)
		Defatted rice bran (DRB)	γ‐Oryzanol	Supercritical CO_2_ extraction of defatted RB with 10% ethanol (w/w) as a co‐solvent at 48.9°C and 23.9 MPa (29.8 g/min CO_2_) yielded 36,000 mg/kg γ‐oryzanol	Kayathi et al. (2021)
	Hot water + ultrasound + EAE	Defatted rice bran (DRB)	Fiber	Hot water, ultrasound, cellulase, and combined hydrolysis‐ultrasound (HC‐US) were compared for rice bran fiber extraction. HC‐US at 50°C for 30 min yielded the highest WS‐DF (9.03 g/100 g) and AS‐DF (9.58 g/100 g) with improved functionality.	Zadeike et al. (2022)
	Supercritical CO_2_ + ethanol‐water co‐solvent	Rice husk (RH)	Total phenolic content Total flavonoid content Antioxidant activity	Supercritical CO_2_ extraction with ethanol‐water (50:50 v/v, 25% w/w) as a co‐solvent at 60°C and 30 MPa for 3 h (1 L/min CO_2_) obtained RH extracts with 1.29 ± 0.09 mg GAE/g TPC, 0.40 ± 0.03 mg CE/g TFC, and 0.23 ± 0.02 mg TE/g antioxidant activity	Kaur and Ubeyitogullari (2023)
	UAE + alkaline hydrolysis	Rice bran (RB)	Bound phenolics	2.5 g bran residue (post‐UAE) + 50 mL of 2 M NaOH, 1 h, acidify to pH 3, extracted with ethyl acetate recovered a sample with DPPH IC50 (55.00 ± 5.37 µg/mL), ABTS IC50 (10.3 ± 0.96 µg/mL), bound phenolics (86.51%), content of FA (369.05 ± 4.91 µg FA/mg)	Guerrini et al. (2020)
	Ultrasound‐assisted + alkaline hydrolysis (UAAH)	Rice bran (RB)	Bound phenolics	Alkaline hydrolysis + ultrasound (30 min) produced ferulic acid (387.36 ± 1.36 µg/mg), bound phenolics (86.87%), content of FA (387.36 ± 1.33 µg FA/mg), DPPH IC50 (38.01 ± 0.52 µg/mL), ABTS IC50 (4.22 ± 0.09 µg/mL)	Guerrini et al. (2020)
	Modified supercritical CO_2_ + (EtOH)	Rice bran (RB)	γ‐Oryzanol Tocopherols Flavonoid Total phenolics FA profile Antioxidant activity	Modified supercritical CO_2_ extraction of rice bran with ethanol (5%–10% w/w relative to CO_2_, 0.40 ± 0.05 kg/h) at 40°C and 40 MPa for 2 h yielded 14.4 g/100 g oil containing 20.63 ± 0.64 mg/g tocopherols, 5.47 ± 0.30 mg/g γ‐oryzanol, 3.42 ± 0.12 mg GAE/g TPC, and 774 ± 6 mg/g total fatty acids	Benito‐Román et al. (2019)
	PEF + UAE + MAE	Black rice	Anthocyanins	Black rice was processed by pulsed electric field (5 kV/cm, 1–3k pulses), microwave (800 W, 20 min), and ultrasonic extraction (60°C, 20 min). PEF and MAE produced the highest total phenolic content, cyanidin‐3‐glucoside/procyanidin‐3‐glucoside, and antioxidant activity	Salee et al. (2023)
	CP (cold plasma) + conventional	Defatted rice bran (DRB)	Phenolic compounds	Cold plasma treatment of DRB at 25°C for 15 min (220 V, ACP) or 20 min (260 V, VCP) under vacuum/atmospheric pressure and the conventional extraction method for treated RB was 75°C for 12 h with 70% ethanol concentration at a ratio of 1:10 increased TPC by 4.66%–7.06%, TFC by 29.11%–43.31%, TFOC by 20.39%–27.28%, and antioxidant capacity by 72.65%–75.65%	Mehta et al. (2022)
		Whole brown rice and brown rice powder	Phenolic compounds Flavonoids Antioxidants Carotenoid	Cold plasma treatment of CPR and CPP at 25 ± 3°C, 160 kV for 3–5 min under 35% ± 5% relative humidity increased TPC to 1273.9 ± 55.4 µg GAE/g DW (CPR) and 1205.8 ± 5.0 µg GAE/g DW (CPP), TFC to 688.4 ± 63.7 µg CE/g DW (CPR) and 1085.8 ± 26.4 µg CE/g DW (CPP), and antioxidant activity (DPPH) to 881.4 ± 9.6 µg Trolox/g DW (CPR) and 766.6 ± 20.2 µg Trolox/g DW (CPP)	Zeng et al. (2023)
	Ultrasound‐assisted extraction + green solvents	Rice bran (RB)	Lipids	Ultrasound‐assisted extraction of rice bran lipids using green solvents, chloroform/methanol (2:1 v/v), methyl *tert*‐butyl ether/methanol (3:1 v/v), water‐saturated 1‐butanol, and ethanol (99%) at 4–20°C and 20 kHz with a 1:20 w/v solid‐to‐solvent ratio achieved lipid yields of 11.7%–19.3%	Guazzotti et al. (2023)
	Ultrasound + cold plasma	Rice bran (RB)	Tocopherols Tocotrienols Sterols Hydrogenated sterols Squalene γ‐Oryzanol Antioxidant activity	Combined ultrasound and cold plasma extraction of rice bran using *n*‐hexane (1:10 w/v) with ultrasound (40°C, 700 W, 20 Hz, 10 min at 40% intensity) followed by cold plasma (30°C, 500 mTorr, 2 kV, 30–45 W, 3 L/min argon, 20 min) achieved an oil yield of 21.66% ± 0.03%	Balegh et al. (2025)
	CO_2_‐expanded hexane extraction	Rice bran (RB)	Rice bran oil (RBO)	CO_2_‐expanded hexane extraction of rice bran at 25°C, 5.1 MPa for 150 min with an optimal CO_2_ mole fraction of 0.88 yielded 0.233–0.250 g oil/g sample, producing phosphorus‐free rice bran oil (<10 ppm)	Mathias et al. (2023)
	Subcritical water–methanol mixture	Rice bran oil (RBO)	γ‐Oryzanol	Subcritical extraction of rice bran oil using a water‐methanol mixture (5/35–35/5 mL/mL; 20/20 mL baseline) at 200°C, 40 bar (N_2_) for 7 h yielded 2750 mg γ‐oryzanol/kg rice bran	Zullaikah et al. (2019)
	SWE + UAE	Rice shell and rice straw	d‐Glucose d‐Xylose d‐Arabinose Cellobiose	Combined subcritical water and ultrasound‐assisted extraction of rice shell and rice straw using distilled water (90–180 g) at 220°C, 25 MPa, and 10 mL/min for 15 min (shell) or 60 min (straw), with ultrasound energy densities of 2.75 × 10^3^ J/cm^3^ (shell) and 5.50 × 10^3^ J/cm^3^ (straw), yielded 14.4 ± 1.5 g/100 g and 13.7 ± 1.6 g/100 g biomass	Rampelotto de Azevedo et al. (2023)
	SHF (separate hydrolysis and fermentation)	Rice straw (RS)	Biobutanol	RS pretreated by microwave‐assisted hydrothermolysis. Separate hydrolysis and fermentation of rice straw using Cellic CTec2 (4.1 FPU/g DW) for enzymatic hydrolysis at 50°C, pH 5.0, 150 rpm for 72 h, followed by fermentation with *Clostridium beijerinckii* (5% v/v) at 37°C, pH 6.4–7.4 for 72 h, produced 4.85 g/L butanol, 7.95 g/L ABE solvents, with a butanol yield of 0.245 g/g sugar, a butanol–biomass ratio of 44.6 g/kg RS, and a productivity of 0.040 g/L h	Valles et al. (2020)
	SSF (simultaneous saccharification and fermentation)	Rice straw (RS)	Biobutanol	RS pretreated by microwave‐assisted hydrothermolysis. Simultaneous saccharification and fermentation of rice straw with Clostridium (5% v/v) using Cellic CTec2 (13.5 FPU/g DW) at 37°C, pH 5.2–6.2 for 48 h with 9% (w/v) biomass loading and 4.7 g/L yeast extract achieved a butanol–biomass ratio of 51 g/kg RS, ABE–biomass ratio of 77 g/kg RS, 5.49 ± 0.09 g/L butanol, and 8.40 ± 0.15 g/L ABE, with a butanol yield of 0.306 ± 0.004 g/g sugar and productivity of 0.114 ± 0.002 g/L h	Valles et al. (2020)
	SSF (s) (solid state fermentation) + enzymatic treatment	Rice husk (RH)	Veratryl alcohol Veratryl aldehyde	Solid‐state fermentation of rice husk with *Phanerochaete chrysosporium* and *Gloeophyllum trabeum* followed by enzymatic treatment using 240 UE/mg lignin peroxidase and 150 UE/mg methoxyl hydrolase at 30°C for 2 h (100 g husk, 40% moisture, citrate buffer 0.2 M, pH 4.5) after 20 days of SSF (20–30°C, pH 5–6) yielded 70.4 ± 0.1 mg/g veratryl alcohol and 23.3 ± 0.3 mg/g veratryl aldehyde	Dias et al. (2023)
	Hydrothermal + enzyme‐assisted extraction	Rice husk and rice straw	Xylooligosaccharides	Hydrothermal treatment of rice husk (170°C, 20 min) and rice straw (180°C, 10 min) at a 1:30 biomass‐to‐water ratio, followed by enzymatic hydrolysis with Pentosane Mono BG (50 U/g for husk, 100 U/g for straw) at 50°C, pH 5.5 for 24 h, achieved 92.17% arabinoxylan conversion with 64.01% XOS yield in husk and 88.34% conversion with 59.52% XOS yield in straw	Jaichakan et al. (2021)
	Extrusion + enzyme	Rice bran (RB)	Soluble dietary fiber Soluble pentosan	Simultaneous extrusion and enzymatic treatment of washed rice bran with xylanase (1%–2% w/w) at screw speeds of 50–100 rpm under barrel (60°C) and die (100°C) temperatures yielded 4% soluble pentosans in warm‐water extracts (37°C)	Dang and Vasanthan (2019)
			Soluble dietary fiber Soluble pentosan	Sequential extrusion and enzymatic treatment of washed rice bran with Bio‐cat xylanase (1%–2% w/w) after extrusion at 100 rpm (60°C barrel, 100°C die) followed by hydrolysis at 55°C for 5 h yielded 6.5% soluble pentosans in warm‐water extracts (37°C) and, in hot‐water extracts (100°C), 25% total soluble with 10.5% soluble pentosans	Dang and Vasanthan (2019)
	SWE + bleaching + acid hydrolysis	Rice husk (RH)	Arabinoxylans Cellulose Nanocrystals Phenolic acids	Subcritical water extraction of milled rice husk at 160°C for 60 min (pH 7) followed by bleaching (80°C, 4 h, acetate buffer 2 M, pH 4.8 with 1.7% chlorite) and acid hydrolysis (45°C, 40 min, 65% H_2_SO_4_) yielded 836.9 ± 13.9 mg/g xylan along with 4.3 ± 0.5 mg/g ferulic acid, 0.4 ± 0.1 mg/g caffeic acid, and 0.8 ± 0.1 mg/g *p*‐coumaric acid	Requena et al. (2019)
Conventional	Soxhlet extraction	Rice bran (RB)	Fatty acids profile Polyphenol Flavonoids γ‐Oryzanol	Soxhlet extraction of rice bran with hexane over 25 cycles yielded 16.3 ± 0.4 g/100 g oil containing 1.58 ± 0.04 mg GAE/g TPC, 1.01 ± 0.09 mg QE/g TFC, 12.47 ± 0.25 mg/g γ‐oryzanol, and a fatty acid profile of 39.4% oleic, 37.7% linoleic, and 15.3% palmitic acid	Benito‐Román et al. (2019)
		Rice bran (RB)	Free fatty acids γ‐Oryzanol	Soxhlet extraction of rice bran with *n*‐hexane for 12 h yielded 17.71% rice bran oil containing 37.71% free fatty acids and 1.33% γ‐oryzanol	Zullaikah et al. (2019)
	Solvent extraction (*n*‐hexane)	Rice bran (RB)	Free fatty acid γ‐Oryzanol	Solvent extraction of rice bran with *n*‐hexane (400 mL) at 69°C for 6 h yielded 18% oil, containing γ‐oryzanol and fatty acids	Baixinho et al. (2025)
	Extrusion	Defatted rice bran (DRB)	Phenolic profiles Bio‐accessibility Antioxidant activity	Extrusion of defatted rice bran at 25% feed moisture, barrel temperatures of 70/98/134°C, and 300 rpm increased free phenolics by 23%, bound phenolics by 50.7%, and total phenolics by 36.3%, enhancing ORAC antioxidant activity (154.5% free, 47.5% bound)	Chen et al. (2019)
		Rice bran (RB)	Soluble dietary fiber Soluble pentosan	Extrusion of washed rice bran at barrel and die temperatures of 60°C and 100°C with screw speeds of 50–100 rpm yielded 4% soluble pentosans in warm‐water extracts (37°C)	Dang and Vasanthan (2019)

Abbreviations: 5‐HMF, 5‐hydroxymethylfurfural; ABTS, 2,2′‐azino‐bis(3‐ethylbenzothiazoline‐6‐sulfonic acid); Bio‐Cat Xylanase, a commercial endo‐1,4‐β‐xylanase; BRB, black rice bran; C3G, cyanidin‐3‐*O*‐glucoside; CE, catechin equivalent; Cellic CTec2, a commercial cellulase enzyme complex (Novozymes) containing cellulases, β‐glucosidases, and hemicellulases; CO_2_‐expanded hexane extraction, a hybrid CO_2_‐expanded solvent process; CP, cold plasma; DPPH, 2,2‐diphenyl‐1‐picrylhydrazyl; DRB, defatted rice bran; EAE, enzyme‐assisted extraction; EC50, half‐maximal effective concentration; EtOH, ethanol; FA PROFILE, fatty acid profile; FA, ferulic acid; FPU, filter paper unit; GAE, gallic acid equivalent; HC–US, hydrolysis combined with ultrasound; IC50, half‐maximal inhibitory concentration; MAE, microwave‐assisted extraction; NaOH, sodium hydroxide; NSI, nitrogen solubility index; ORAC, oxygen radical absorbance capacity; PEF, pulsed electric field; Pentosane Mono BG, a commercial xylanase preparation derived from *Aspergillus oryzae*; PLE, pressurized liquid extraction; QE, quercetin equivalent; RB, rice bran; RBO, rice bran oil; RE, rice endosperm; RF, rice fiber; RH, rice husk; RS, rice straw; SC‐CO_2_, supercritical carbon dioxide; SC‐CO_2_, supercritical carbon dioxide extraction; SFE, supercritical fluid extraction; SHF, separate hydrolysis and fermentation; SSF (liquid), simultaneous saccharification and fermentation (liquid system); SSF (s), solid‐state fermentation; SSF, solid‐state fermentation; SWE, subcritical water extraction; TE, Trolox equivalent; TFC, total flavonoid content; TFOC, total flavanol content; TPC, total phenolic content; UAAH, ultrasound‐assisted alkaline hydrolysis; UAE, ultrasound‐assisted extraction; UE, enzyme unit; UFAs, unsaturated fatty acids; UFAs/SFAs, unsaturated/saturated fatty acids; XOS, xylo‐oligosaccharides.

Despite aligning with the principles of green chemistry, CP faces several industrial challenges and limitations. High equipment costs, issues with scalability, and difficulties in achieving uniform treatment at larger volumes represent significant barriers. Moreover, excessive generation of reactive oxygen and ROS/RNS can lead to oxidative degradation of sensitive molecules (Misnal et al. 2022; Zhou et al. 2025). None of the cited research studies reported quantitative capital expenditure (CAPEX), operational expenditure (OPEX), energy consumption per unit mass, throughput capacity, LCA, or techno‐economic analysis (TEA). Published manuscripts emphasize that discharge uniformity and penetration depth limitations are decisive challenges for industrial implementation (Wagner et al. 2023). Consequently, although CP demonstrates clear mechanistic and functional advantages for enhancing the extraction and stabilization of RB and related matrices, its technological readiness remains constrained by engineering scalability and economic validation. To address these challenges, future efforts should focus on optimizing reactor designs, integrating complementary nonthermal techniques, and conducting comprehensive techno‐economic and LCAs to establish CP as a commercially viable extraction technology (Balegh et al. 2025).

### 4.2 Enzyme‐Assisted Extraction

EAE, also known as aqueous enzymatic extraction (AEE), has emerged as a versatile biocatalytic approach for the eco‐efficient recovery of high‐value biomolecules from RB, RH, RS, and rice germ. By utilizing hydrolytic enzymes such as cellulases, pectinases, proteases, xylanases, and amylases, EAE effectively disrupts the rigid structures of plant cell walls and protein–polysaccharide complexes. This process facilitates the release of oils, proteins, phenolics, polysaccharides, and dietary fibers under mild physicochemical conditions while minimizing environmental impact and eliminating the need for organic solvents (Koysuren et al. 2021; Kumar et al. 2023; Marathe et al. 2019). By operating within the temperature range of 35–60°C and tailored pH levels for each enzyme, EAE prevents thermal degradation, eliminates toxic solvents, and improves extraction selectivity (Fraterrigo Garofalo et al. 2021; Huang et al. 2024). In detail, the extraction time is moderate to long (hours), with medium cost associated with enzyme procurement and temperature control. The yield is typically moderate to high, especially for proteins and phenolics from RB. Extraction efficiency is significantly impacted by various parameters, including enzyme concentration, enzyme/substrate ratio, particle size, incubation time, and pH (Chemat et al. 2020; Krakowska‐Sieprawska et al. 2022). Reducing particle size through grinding enhances substrate accessibility and accelerates hydrolysis. This type of extraction was first applied to the recovery of RBO by Sengupta and Bhattacharyya (1996), who used pectinase and cellulase to evaluate the influence of temperature, reaction time, and enzyme and RB concentrations. The results showed that this technique makes it possible to obtain oil of high quality comparable to that produced by conventional solvent extraction, allowing also the fractionation of different components from the RB, such as proteins and lipids. However, EAE alone does not guarantee high extraction yields. For this purpose, during the following years, several studies (Hernandez et al. 2000; Sharma et al. 2001) have tested combinations of commercial enzymes, such as proteases, α‐amylases, and cellulases, which act by degrading cell walls and increasing the permeability of lipid membranes. Sharma et al. (2001) find that under optimal conditions (about 65°C, pH 7, and prolonged agitation), it was possible to obtain yields corresponding to 76%–78% of bioactive compounds compared to those achieved with conventional extraction methods, without the use of organic solvents.

Moreover, enzyme cocktails demonstrate superior performance compared to single enzymes due to their synergistic effects. For example, a mixture of cellulase, pectinase, xylanase, and protease have been shown to achieve recovery rates of up to 92% for RBO, along with notable increases in γ‐oryzanol and antioxidant phenolics, such as ferulic and protocatechuic acids (Cheetangdee 2019; Tufail et al. 2024; Xu et al. 2021). Similarly, Saveboworn et al. (2025) added RB protein (RBP) extract obtained from EAE using alcalase and Flavourzyme enzymes to a fruit juice beverage to create a new high‐nutritional‐value alternative for consumers.

The specific applications of enzymes underscore the versatility of EAE. Amylases release starch‐bound proteins and enhance lipid recovery, whereas cellulases hydrolyze β‐1,4‐glycosidic linkages in cellulose, thereby liberating proteins and lipids. Pectinases disrupt the middle lamella to facilitate the release of oils and phenolic compounds. Proteases break down protein matrices to produce bioactive peptides that possess antioxidant and antihypertensive properties, and xylanases increase soluble dietary fiber (SDF), yielding prebiotic xylo‐oligosaccharides with conversion rates of approximately 88% from RS arabinoxylan (Dang and Vasanthan 2019; Jaichakan et al. 2021). Protein hydrolysates derived from EAE exhibit improved solubility, emulsifying and foaming characteristics, as well as angiotensin‐converting enzyme (ACE) inhibitory activity (Liu et al. 2019; Rodríguez‐Restrepo et al. 2020). When applying alcalase‐assisted EAE to RB, a yield of 79% RBO was obtained, with γ‐oryzanol concentrations ranging from 1.76 to 2.43 g/100 g of oil (Hanmoungjai et al. 2001). The oil obtained showed a composition similar to that one obtained by solvent extraction, but with lower FFA levels, although slightly higher peroxide values. Furthermore, alcalase enzyme proved to be particularly effective compared to other enzymes like Celluclast, hemicellulase, Pectinex Ultra SP‐L, Viscozyme L, and papain, allowing yields of about 75% to be reached in just 1 h of reaction as noticed by Hanmoungjai et al. (2002). Meanwhile, a xylanase‐plant enzyme cocktail achieved a yield of 76.95% RBO (Xu et al. 2021) compared to 72.97% and 73.27% yield from plant‐extracted enzyme and xylanase, respectively. Furthermore, multienzyme hydrolysis, performed using glucoamylase, acid cellulase, and acid protease, significantly increased total phenolic and flavonoid contents by 46.24% and 79.13%, respectively, in the RB extract, releasing compounds such as ferulic acid, protocatechuic acid, and quercetin with consequent increases in the antioxidant activities measured with FRAP and ORAC assays (159.14% and 41.98%, respectively) (Liu et al. 2017; Huang et al. 2024).

Despite its advantages, EAE encounters several limitations. Key challenges include the high cost of commercial enzymes, incomplete hydrolysis due to the complexity of the matrix, emulsion formation during oil recovery, and difficulties in scaling up the process (Brondani Teixeira Ribas et al. 2025; Jafarzadeh et al. 2025). In addition, extraction using this technique generally requires longer processing times than conventional methods, and enzyme activity is highly sensitive to process conditions such as pH and temperature, requiring precise control to maintain efficiency (Sowbhagya and Chitra 2010). Moreover, purification represents a critical step that increases process complexity and overall production costs (Nadar et al. 2018). To facilitate industrial applications, research into immobilized, recombinant, or engineered enzymes, as well as strategies for process intensification, is essential. Additionally, extrusion pretreatment has demonstrated its ability to restructure the RB matrix, thereby improving enzyme accessibility and enhancing the efficiency of subsequent EAE processes (Modupalli et al. 2024). Complementary studies further highlight the nutritional benefits of enzymatic modification. Dang and Vasanthan (2019) showed that enzymatic treatment with four different xylanase enzymes increased the SDF content of washed RB, whereas Zadeike et al. (2022) reported that enzymatic treatment with cellulase enzyme of DRB enhanced the porosity of water‐soluble fiber and promoted the formation of a more homogeneous alkali‐soluble fiber network (Table 2).

### 4.3 Microwave‐Assisted Extraction

MAE has emerged as a highly innovative and sustainable technology for recovering valuable compounds from rice by‐products, particularly RB. Its advantages, including speed, reduced solvent usage, and high extraction efficiency, position it as a compelling alternative to conventional extraction techniques (Das et al. 2025; Fraterrigo Garofalo et al. 2021; Huang et al. 2024; Modupalli et al. 2024). Extraction time is fast (minutes) with medium equipment cost. Yield can be high, but thermal exposition may degrade sensitive compounds if not carefully controlled. MAE is effective for polar metabolites, though prolonged exposure may reduce thermolabile nutrient content during the processing. This technique also presents some limitations link to nonuniformed heating with the risk of degradation of thermosensitive compounds (Mandal et al. 2007). Microwaves function within the frequency range of 0.3–300 GHz, with 2.45 GHz and 915 MHz being the most commonly utilized in industrial applications (Anyiam et al. 2025; Chemat et al. 2020). The heating process occurs through dipolar rotation, where polar molecules oscillate at frequencies of approximately 2.45 × 10^9^ s^−1^, and ionic conduction, which involves ion migration that generates resistive heating. These mechanisms lead to rapid volumetric heating that increases intracellular pressure, disrupts cell walls, and enhances the diffusion of bioactive compounds into solvents (Kumar et al. 2023; M. Ramos et al. 2023). Solvent selection is a crucial factor influencing the efficiency of MAE. Polar solvents, including ethanol, methanol, and water, are particularly effective at absorbing microwaves. Ethanol–water mixtures are often reported as optimal for extracting γ‐oryzanol, tocopherols, and phenolics, all while adhering to sustainability principles (Arellano et al. 2022; Fărcaș et al. 2022; Xi et al. 2024). Ethanol is preferred as a green, food‐grade solvent, whereas methanol, while capable of achieving very high yields (approximately 96% extractability and about 80% higher phenolic recovery), is limited in food applications due to its toxicity (Modupalli et al. 2024). Under subcritical conditions (100–374°C, 10–80 bar), water experiences a reduction in polarity, facilitating the solubilization of semipolar metabolites such as anthocyanins (Carpentieri et al. 2021).

Optimization studies indicate that microwave powers ranging from 500 to 1000 W, extraction temperatures between 80°C and 120°C, solid‐to‐liquid ratios of 1:3, and treatment times varying from seconds to approximately 90 min yield effective extraction conditions for RB (Ali et al. 2026; Modupalli et al. 2024). Pilot‐scale systems have demonstrated strong scalability, with continuous MAE achieving approximately 90% recovery of RBO in just 8 min using ethanol (Terigar et at. 2011). Comparative studies reveal that MAE not only outperforms Soxhlet extraction in terms of efficiency and speed but also competes favorably with UAE and SFE regarding yield and preservation of bioactives. For instance, a two‐step MAE process achieved over 95% recovery of RBO enriched in phospholipids and tocopherols while simultaneously extending shelf life through the inactivation of lipase. The rapid heat generation from microwave irradiation denatured the enzyme, preventing the hydrolysis of triacylglycerols into FFAs. This stabilization allowed the bran to be stored for extended periods without significant deterioration, typically keeping FFA levels below 5% for over 100 days (Arellano et al. 2022; Pinto et al. 2021). Similarly, Frosi et al. (2023) reported that MAE of RH using 80% aqueous ethanol at 500 W for short extraction times yielded extracts rich in polyphenolic compounds. San Sebastián et al. (2026) investigated the effects of temperature, extraction time, and solvent ratio while maintaining the microwave power at 500 W on RS. By using a response surface methodology, the optimal MAE conditions were determined. Under these conditions, RS extracted with 50 mL of 60% (v/v) ethanol at 80°C for 40 min and 500 W yielded the best extraction efficiency (18.9%), with a high TPC of 796.1 mg gallic acid equivalent/100 g. Furthermore, the MAE extract exhibited strong antioxidant activity, as measured by FRAP and ABTS assays, with values of 32.3 µmol Trolox equivalent/g and 43.2 µmol Trolox equivalent/g, respectively. Beyond extraction, microwave treatment has also been widely investigated for RB stabilization. Aluthge et al. (2023) demonstrated that microwaved RB exhibited elevated levels of total phenolics and γ‐oryzanol in the recovered oil, whereas Lavanya et al. (2019) confirmed that microwave stabilization suppressed enzymatic activity, thereby reducing rancidity. In addition to enhancing stability, microwave heating has been shown to improve the techno‐functional properties of RB. Irakli et al. (2020) observed enhanced foaming capacity, emulsifying properties, and water absorption following microwave treatment (Table 2). The combination of UAE and MAE on the black RH was studied by Jha et al. (2017) using response surface methodology. Different factors were analyzed: sonication time and temperature, solid/solvent ratio, and microwave time. Under the best conditions, 10 min of sonication at 50°C, with solid/solvent ratio of 1:41, ethanol percentage of 67, and 31 s of microwave time, it was possible to obtain an extract with the highest content of flavonoids and anthocyanins (3.01 mg and 3.36 mg/100 g, respectively), 1.72 mg/g gallic acid equivalents of phenolic compounds, and 100% antioxidant activity.

### 4.4 Ohmic‐Assisted Extraction

OHAE, also known as electroconductive or Joule heating, is a nontraditional thermal technology that is gaining attraction for the valorization of rice by‐products. Processing time is moderate, energy consumption is medium, and yield can be relatively high. The uniform heating reduces local overheating, preserving heat‐sensitive metabolites. Ohmic methods are particularly suited for liquid‐rich matrices, maintaining antioxidant and nutrient integrity throughout processing. This method involves passing alternating current through biomass, which results in rapid and uniform volumetric heating while simultaneously inducing electroporation of cell membranes. This process enhances the diffusion of intracellular bioactives into solvents (Khalid et al. 2024; Talha et al. 2024). Heat is generated as ions migrate toward electrodes, dissipating energy resistively, whereas electric fields increase membrane permeability by creating pores that improve extractability (Khalid et al. 2024; Modupalli et al. 2024; Talha et al. 2024). In comparison to conventional heating, OHAE reduces the occurrence of localized hot spots, preserves thermolabile compounds, and enhances both the yield and quality of extracts (Buljeta et al. 2023; Modupalli et al. 2024). This technique improves cell permeability and mass transfer, thereby reducing extraction time, energy consumption, and solvent usage while preserving heat‐sensitive bioactive compounds (Sakr and Liu 2014). The performance of this process is significantly influenced by various operational parameters, including electric field strength (EFS), voltage, waveform, treatment duration, and the properties of the sample, such as moisture content and conductivity. Despite its advantages, OHAE is limited by high initial equipment costs, electrode corrosion, and nonuniform heating in heterogeneous materials, which can reduce extraction efficiency and reproducibility (Sakr and Liu 2014). For RB, optimal conditions typically involve an EFS ranging from 50 to 225 V/cm at a frequency of 50–60 Hz, with moisture levels adjusted to 30%–40% to improve conductivity and efficiency (Yılmaz Tuncel 2023). Optimal enzyme‐assisted ohmic heating extraction has been reported to occur at approximately 54°C for 5 min with an electrical field strength of 179 V/cm, resulting in over 80% recovery of RBO (Khalid et al. 2024). Under these conditions, lipase activity is effectively inhibited by the rapid heating and electrical effects (electroporation). Untreated RB accumulated around 41% FFAs after 75 days of storage, whereas ohmically treated RB maintained FFA levels at just 4.7%, with additional stabilization observed during cold storage (Das et al. 2025; Liu et al. 2019). In addition to stabilization, OHAE enhances the recovery and retention of bioactive compounds. Research on black RB has shown significantly higher preservation of anthocyanins, including cyanidin‐3‐*O*‐glucoside, delphinidin, pelargonidin, and malvidin, compared to steam‐based extraction methods (Talha et al. 2024; Zhou et al. 2025). Furthermore, OHAE has been effectively utilized to extract pectin, essential oils, proteins, cellulose fibers, and fruit juices, consistently outperforming conventional thermal processes in terms of yield, extraction time, and energy efficiency (Chemat et al. 2020; Jafarzadeh et al. 2025; Manzoor et al. 2023).

### 4.5 Pressurized Liquid Extraction

PLE, also known as ASE or enhanced solvent extraction (ESE), is a highly efficient solid–liquid separation technology increasingly applied for the recovery of high‐value compounds from rice by‐products. Common solvents used in PLE include water, ethanol, or their mixtures. Among these, water is considered one of the most promising solvents due to its environmental compatibility. The method typically operates at elevated temperatures (50–250°C) and pressures (4–20 MPa), conditions that maintain solvents in the liquid state above their atmospheric boiling points and increase the efficiency of extraction. Under these parameters, solvent viscosity and surface tension decrease, whereas diffusion coefficients and mass transfer rates increase, collectively enhancing solvent penetration and the rapid desorption of intracellular compounds such as phenolics, oils, and proteins (Cheetangdee 2019; Frosi et al. 2023; Gil‐Martín et al. 2022; Kumar et al. 2023). Extraction time is short to moderate, but the equipment cost is quite high. Yield is very high for both polar and nonpolar compounds. Although heat‐tolerant compounds are efficiently extracted, thermolabile metabolites may suffer degradation, careful temperature optimization is required to protect bioactive components during processing. These problems can be resolved by doing careful process optimization (Mustafa and Turner 2011). Compared to conventional methods such as Soxhlet or maceration, PLE significantly reduces solvent use and shortens extraction times to as little as 5–20 min, while achieving recovery rates approaching 95%–100% in many systems (Gil‐Martín et al. 2022; Usman et al. 2023). The technique is compatible with food‐grade solvents such as water and ethanol, making it particularly attractive for nutraceutical applications. Echenique et al. (2024) showed that ethanol‐based PLE operated in an intermittent (cyclic) regime efficiently recovers RBO while maintaining acceptable quality attributes. Leonarski et al. (2023) demonstrated that continuous‐flow PLE with an acidified ethanolic solvent yields anthocyanin‐rich extracts from black RB with enhanced antioxidant performance and cytocompatibility. Ramos et al. (2024) confirmed that intermittent ethanol‐PLE enables efficient RBO extraction while markedly reducing solvent consumption compared with semicontinuous operation (Table 2).

Commercial PLE systems offer programmable operation, improving reproducibility and scalability. However, several limitations constrain its application. Co‐extraction of undesirable compounds, dilution of extracts during multi‐cycle runs, and relatively high equipment costs remain significant challenges. Moreover, if not carefully optimized, high temperatures and pressures can accelerate the degradation of thermolabile compounds, reducing the nutritional and functional quality of extracts (Kumar et al. 2023). Despite its eco‐friendly profile, PLE faces challenges at scale, including equipment corrosion under high temperatures, the need for precise optimization of temperature–pressure conditions, and the high energy demand of water evaporation steps. To address these barriers, hybrid systems integrating PLE with UAE, EAE, or SFE have been explored, offering improved selectivity and expanded recovery profiles. Such innovations position PLE as a cornerstone of integrated green biorefineries aimed at sustainable valorization of rice by‐products (Modupalli et al. 2024).

### 4.6 Pulsed Electric Field Extraction

PEF extraction is an innovative nonthermal green technology that utilizes short, high‐voltage electrical pulses (ranging from 0.1 to 80 kV/cm and lasting microseconds to milliseconds) to treat plant tissues. This technique induces electroporation of cell membranes, resulting in increased membrane permeability, decreased diffusion resistance, and enhanced solvent penetration. Consequently, it promotes the effective release of intracellular compounds (Athanasiadis et al. 2023; Chatzimitakos et al. 2023; Kumar et al. 2023). The degree of permeabilization is influenced by several factors, including field strength, pulse duration, frequency, total energy input, and the characteristics of the solvent, such as pH and conductivity (Carpentieri et al. 2021). Treatment time is extremely short (seconds), with medium equipment cost. Yield is generally high, and nonthermal operation preserves thermolabile compounds, such as phenolics and vitamins. PEF‐treated RB maintains metabolite integrity during subsequent processing, supporting functional food applications. The implementation of PEF is limited by high equipment cost and the complex process optimization but also by low extraction yield with dense or highly conductive materials due to the difficulty to disintegrate the cell matrix (Barba et al. 2015). In rice‐based systems, PEF treatment has shown significant enhancements in both yield and functional quality. For brown rice, applying PEF at 2 kV/cm with 1000 pulses resulted in a 50% increase in antioxidant activity and elevated levels of chlorogenic and ferulic acids, γ‐oryzanol, and other phenolic compounds (Quagliariello et al. 2016). Thongkong et al. (2023) reported that in RB, PEF at 2.3 kV/cm led to a protein yield increase of approximately 21% compared to traditional alkaline extraction, while maintaining the integrity of amino acid profiles. Notably, structural modifications toward more ordered protein conformations were observed, which correlated with improved emulsifying and foaming properties as well as enhanced digestibility. Furthermore, PEF reduced lipase activity in RBO by around 40% causing electroporation of membranes, which can lead to structural damage in lipase enzymes, thus significantly extending RBO oxidative stability during storage (Modupalli et al. 2024). Mechanistic evidence highlights electroporation as the primary mechanism at work. Scanning electron microscopy (SEM) demonstrates the formation of pores in the cell walls of RB subjected to PEF treatment, whereas such pore formation is absent in untreated controls (Thongkong et al. 2023). Beyond rice, PEF has been successfully applied to a range of agri‐food by‐products, facilitating the extraction of valuable compounds such as polyphenols, flavonoids, anthocyanins, and pectins from sources like citrus peels, grape stems, and jackfruit waste. These extraction methods generally require lower volumes of solvents and reduced energy inputs compared to traditional techniques (Chatzimitakos et al. 2023; Ran et al. 2019).

### 4.7 Subcritical Fluid/Water Extraction

SWE is a specific form of PLE in which water is used as the sole extraction solvent under subcritical conditions (100–374°C). In this process, pressure primarily serves to keep the water in its liquid state, while elevated temperature significantly alters its physicochemical properties, particularly the dielectric constant. As a result, water behaves as a less polar solvent, enabling the efficient extraction of a wide range of bioactive compounds without organic solvents. SWE therefore allows high extraction efficiency, reduces environmental impact, and preserves the quality of sensitive compounds (Krivošija et al. 2025). SWE significantly increases both yield and functionality of extracts; however, challenges such as high energy demands, corrosion, and the degradation of sensitive compounds continue to limit its application (Buljeta et al. 2023; Wongthaweewatana et al. 2021). Extraction time is moderate, but equipment cost is high due to pressure levels. The yield can be relatively high, but heat‐sensitive metabolites may degrade at elevated temperatures. SbFE and SWE are gaining recognition as environmentally sustainable and highly selective methods for valorizing rice by‐products (Das et al. 2025; Modupalli et al. 2024; Requena et al. 2019). SbFE utilizes solvents such as propane, butane, ethanol, or CO_2_, all maintained below their critical points and typically under moderate pressures of less than 10 MPa. These conditions facilitate the efficient extraction of lipophilic compounds, particularly oils and phenolics. For instance, blends of propane and butane have been demonstrated to yield high‐quality RBO enriched with γ‐oryzanol and tocopherols while also exhibiting a lower FFA content compared to traditional hexane extraction methods (Modupalli et al. 2024). With SWE, at elevated temperatures, water demonstrates a lower dielectric constant and viscosity, alongside an increased ionic product, which enhances its ability to dissolve semipolar compounds and facilitate hydrolytic reactions and imparts weak acid‐base catalytic activity (Buljeta et al. 2023; Lee et al. 2025). Moreira et al. (2023) demonstrated that in RB, SWE has been effectively employed for the simultaneous inactivation of lipase and stabilization of oil, in addition to generating up to 6.5 g of fermentable sugars per 100 g of DRB, thereby supporting biofuel production applications. Indeed, the high temperatures used in SWE (typically between 120°C and 240°C) provide sufficient thermal energy to disrupt the hydrogen bonds and hydrophobic interactions that maintain the lipase's three‐dimensional structure. Moreover, short residence times (≤5 min) result in the production of antioxidant‐rich hydrolysates with prebiotic potential and unique sensory attributes (Lee et al. 2025). SWE has also been applied to RH, where mild conditions (180–200°C) facilitate the recovery of arabinoxylans containing ester‐linked phenolics, followed by the isolation of cellulose nanocrystals and biosilica (Requena et al. 2019). In addition, Wongthaweewatana et al. (2021) employed SWE to obtain protein hydrolysates and plant‐based milk analogues from DRB, demonstrating its promise in sustainable food formulations (Table 2).

Although SWE is particularly effective for extracting polar and semipolar bioactives, high temperatures may lead to the degradation of thermolabile constituents, resulting in the formation of by‐products such as furfural and hydroxymethylfurfural (Colombo et al. 2024). Additional challenges include equipment corrosion, high capital costs, and the necessity for post‐extraction concentration steps, which hinder large‐scale implementation. To address these issues, various process intensification strategies have been investigated, including UAE‐ or EAE SWE, as well as integration with CP or PEF, both of which enhance the recovery of sugars and phenolics while minimizing thermal stress (Balegh et al. 2025; Rampelotto de Azevedo et al. 2023).

### 4.8 Supercritical Fluid Extraction (SFE)

SFE is widely recognized as one of the cleanest and most versatile green technologies for recovering bioactive compounds from rice by‐products, avoiding solvent residues and preserving nutritional and functional properties. Supercritical carbon dioxide (SC‐CO_2_) serves as the predominant solvent in food and nutraceutical applications, thanks to its moderate critical point (31.1°C, 7.38 MPa), nontoxicity, and ease of removal following depressurization (Chemat et al. 2020; Colombo et al. 2024). Extraction time is moderate, with high equipment and operational costs. Yield for oils, sterols, and other lipophilic metabolites is high, and the nonthermal nature preserves sensitive compounds. CO_2_ extraction ensures minimal oxidation and is compatible with maintaining nutrient quality through processing. The equipment of this extraction technique is the main crucial factor and the limitation of its use. Moreover, this system allows the extraction of lipophilic compounds, reducing the efficiency of polar compound extraction if cosolvents are not use, such as ethanol. SC‐CO_2_ effectively extracts nonpolar lipophilic compounds such as γ‐oryzanol, tocopherols, tocotrienols, sterols, and unsaturated fatty acids, resulting in oils that exhibit superior oxidative stability when compared to traditional hexane extraction (Das et al. 2025; Huang et al. 2024). With optimized operating conditions (40°C, 30 MPa), obtained using a response surface methodology, Wang et al. (2008) recovered 18.1% of oil from RB yield with γ‐oryzanol extraction efficiency of 88.5%, alongside significantly enhanced antioxidant profiles. Other authors (Tomita et al. 2014) studied the correlation between different parameters such as pressure and temperature. They observed that at higher pressures, increases in temperature produced only small changes in RBO recovery. In contrast, a significant decrease in oil recovery was observed when the temperature increased from 40°C to 80°C. Moreover, at high pressures the solubility of the oil decreases, which consequently leads to a reduction in yield.

Due to the limited affinity of SC‐CO_2_ for polar compounds, co‐solvents, such as ethanol or ethanol–water mixtures, are often added to enhance polarity and broaden selectivity (Benito‐Román et al. 2019; Kaur and Ubeyitogullari 2023). Ethanol‐modified SC‐CO_2_ has been shown to achieve higher recoveries of γ‐oryzanol and phenolic compounds compared to Soxhlet extraction, while the addition of water improves hydrogen bonding and porosity effects in RB and RH matrices. Furthermore, hybrid processes enhance extraction efficiency; for instance, ultrasound‐assisted supercritical fluid extraction (UA‐SFE) has demonstrated up to a 35% increase in yields and a 60% reduction in processing time (Deng et al. 2022; Soares et al. 2018). Beyond SC‐CO_2_, supercritical water extraction (SCWE) operating at temperatures exceeding 374°C and pressures above 22.1 MPa facilitates the hydrolytic depolymerization of RH, RS, and RB. This process aids in the recovery of valuable compounds, such as phenolic acids, short peptides, nanocellulose, and silica (Requena et al. 2019; Wongthaweewatana et al. 2021). Additionally, alternative methods like CO_2_‐expanded liquids, such as CO_2_‐hexane systems, have been investigated, providing milder operating conditions, higher yields, and lower levels of FFA and phosphorus in extracted oils (Huang et al. 2024; Mathias et al. 2023). Pinto et al. (2021) showed that SC‐CO_2_ effectively extracts RBO enriched in unsaturated fatty acids, with linoleic and oleic acids predominating, thereby conferring strong antioxidant activity. Pilot‐scale applications have further validated its industrial feasibility. Wang et al. (2008) and Baixinho et al. (2025) reported that SC‐CO_2_ processing of RB produced γ‐oryzanol‐enriched oils under relatively low pressures and temperatures, achieving high extraction yields at larger scales. Complementarily, Moreira et al. (2023) applied SC‐CO_2_ to extract RBO, enabling subsequent valorization of the DRB via subcritical water hydrolysis (Table 2).

### 4.9 Ultrasound‐Assisted Extraction

UAE has emerged as one of the most extensively researched green technologies for the valorization of rice by‐products, such as RB, RH, RS, and rice germ. The extraction process offers a short extraction time, medium equipment cost, reduced energy, and solvent requirements, achieving high yields, particularly for phenolics and proteins. Mild temperature conditions help to preserve thermolabile compounds. Nevertheless, it presents limitations such as difficulties in nonuniform cavitation effects and potential degradation of sensitive compounds due to localized heating and free radical formation (Chemat et al. 2017). UAE utilizes acoustic waves in the frequency range of 20–100 kHz to create alternating cycles of compression and rarefaction within liquid–solid matrices. These cycles generate cavitation bubbles that collapse with great intensity, producing localized hot spots exceeding 5000 K and high‐pressure microjets of over 1000 atm. The physical effects of this process disrupt plant cell walls, reduce particle size, and improve solvent penetration, which, in turn, accelerates mass transfer and facilitates the extraction of valuable compounds like oils, proteins, phenolics, polysaccharides, and γ‐oryzanol (Chemat et al. 2020; Modupalli et al. 2024; Tufail et al. 2024).

In RB, UAE has significantly reduced extraction times from hours to mere minutes while simultaneously enhancing oil quality by minimizing oxidation and preserving valuable tocopherols and phytosterols. The first study was made by Cravotto et al. (2004) using high intensity ultrasounds (300 W, at 45°C for 30 min) obtaining 21% of oil. The optimized operating conditions typically involve ultrasonic frequencies ranging from 20 to 40 kHz, power densities between 100 and 500 W, extraction times of 5–30 min, and the use of ethanol or ethanol‐water mixtures as solvents (Arellano et al. 2022; Fraterrigo Garofalo et al. 2021). Tabaraki and Nateghi (2011), using response surface methodology, optimized the extraction of oil, polyphenols, and antioxidant compounds from RB. Three key process variables were evaluated: ethanol concentration, extraction time, and temperature. The highest oil extraction yield was obtained with 28 min of sonication in an ultrasonic bath operating at 35 kHz and 140 W at a temperature of 60°C using an ethanol concentration of 87%. Beyond oil recovery, UAE has demonstrated remarkable efficacy in isolating protein hydrolysates that exhibit improved solubility and ACE inhibitory activity, as well as polysaccharides and dietary fibers with enhanced prebiotic functionality (Liu et al. 2019; Ran et al. 2019; Rodríguez‐Restrepo et al. 2020). In pigmented RB, Bunmusik et al. (2023) reported that solvent‐based UAE maximized the extraction of bioactive phytochemicals. Similarly, Guerrini et al. (2020) demonstrated that UAE yielded the highest levels of free phenolic compounds from RB. The structural effects of ultrasound have also been investigated. Zadeike et al. (2022) showed that ultrasound pretreatment of DRB significantly altered dietary fiber polysaccharides by disrupting covalent linkages, resulting in a cracked and more porous capillary structure.

### 4.10 Hybrid and Integrated Extraction Approaches

Hybrid extraction systems that integrate two or more green technologies have emerged as highly effective strategies for valorizing rice by‐products. The key rationale behind these systems is the complementarity of their mechanisms. UAE enhances solvent penetration and disrupts cellular structures, whereas PLE and SWE improve solubilization under elevated temperatures and pressures. Additionally, techniques, such as CP, PEF, and OHAE, facilitate nonthermal membrane permeabilization (Das et al. 2025; Modupalli et al. 2024; Xi et al. 2024).

Several integrated approaches have demonstrated significant improvements in rice processing. The combination of UAE with PLE or SWE has been shown to enhance the recovery of phenolic compounds and γ‐oryzanol while simultaneously reducing processing times by 40%–60% compared to using individual methods (Requena et al. 2019). Similarly, the integration of MAE with UAE has led to increased recovery of RBO, improved retention of phospholipids, and reduced lipase activity, surpassing the performance of either technique in isolation. The inactivation of lipase during the combined MAE and UAE occurred through a synergistic process of denaturation and structural disruption (Fraterrigo Garofalo et al. 2021; Pinto et al. 2021). EAE with UAE has further enhanced protein hydrolysis efficiency and oil release, producing hydrolysates that are enriched in antioxidant peptides and phenolic compounds (Jafarzadeh et al. 2025; Tufail et al. 2024). In ultrasound‐assisted enzyme extraction (UAEE), protein recovery from brewer's spent grain reached an impressive 86.16%, whereas the oxidative stability and antioxidant activity of RBO were significantly enhanced compared to conventional methods (Zaky et al. 2022). The combination of UAE or PEF with SFE has enhanced CO_2_ penetration and improved mass transfer, resulting in oil yields that are up to 35% higher than those achieved with conventional SFE, all while preserving the integrity of tocopherols and sterols (Soares et al. 2018; Wei et al. 2016). Similarly, the integration of SWE with either CP or PEF has facilitated the recovery of fermentable sugars and phenolic‐rich extracts, minimizing thermal degradation (Balegh et al. 2025). Additionally, sequential treatments involving CP and UAE have demonstrated strong synergistic effects. For example, in wood apple shell, a combination of CP at 20 s and UAE at 15 s increased the total phenolic content from 24.57 ± 1.89 to 63.84 ± 0.36 mg GAE/g DW, alongside heightened concentrations of flavonoids and enhanced antioxidant activity (Balegh et al. 2025; Murakonda and Dwivedi 2024). Zadeike et al. (2022) reported that high‐pressure homogenization combined with ultrasound (HC + US) yielded RB fractions with high purity and uniform porous structures. Sequential extrusion‐enzyme treatments notably increased SDF content by fourfold, particularly pentosan fractions (Dang and Vasanthan 2019). Microwave plus steaming stabilization was reported by Aluthge et al. (2023) as the most effective approach for maximizing oil extractability and oxidative stability in RB. Ultrasound‐heat treatment (USHT; 30 min US + 60 min HT) achieved the highest total phenolic content in RS as described by Freitas et al. (2020). Mathias et al. (2023) reported that hybrid CO_2_‐expanded systems, such as CO_2_‐hexane extraction, produced high‐quality RBO with improved bioactive retention, whereas modified SC‐CO_2_ with ethanol co‐solvent enhanced antioxidant activity and γ‐oryzanol content, yielding oil of superior quality (Benito‐Román et al. 2019; Kayathi et al. 2021). Rampelotto de Azevedo et al. (2023) revealed that UAE combined with SWE added value to agricultural residues via renewable pathways. Moreover, SC‐CO_2_ consistently enabled the recovery of γ‐oryzanol‐enriched RBO under mild temperature and pressure, ensuring product stability while avoiding toxic solvents (Table 2) (Benito‐Román et al. 2019; Fraterrigo Garofalo et al. 2021).

Collectively, these studies underscore the considerable advantages of integrated and sequential processing strategies, which enhance extraction efficiency, preserve bioactive compounds, and improve functional and structural properties of rice‐based matrices. From a sustainability standpoint, hybrid approaches resonate with the principles of a circular economy. They enhance selectivity, minimize solvent and energy consumption, and facilitate the cascade recovery of various fractions, such as lipids, proteins, polysaccharides, phenolics, and minerals, from a single biomass stream (Anyiam et al. 2025; Buljeta et al. 2023). However, the wider adoption of these methods is hindered by high equipment costs, operational complexity, and the absence of standardized protocols. To support industrial‐scale implementation, future efforts should focus on techno‐economic evaluations, reactor design optimization, and multi‐scale modeling.

## 5 Rice By‑Products Applications

The utilization of rice by‐products in food applications follows two main and complementary approaches: the direct incorporation of whole or stabilized by‐products (e.g., after the inactivation of lipase enzyme) and the use of extracted or modified fractions as functional ingredients. Although extraction processes enable the isolation and concentration of specific bioactive compounds, such as proteins, lipids, and phenolics, enhancing their functionality and enabling targeted applications, the direct use of by‐products (e.g., RB, DRB, or RH flour) remains highly attractive from a technological, economic, and sustainability perspective. In many cases, minimal processing (e.g., stabilization, milling, or defatting) is sufficient to preserve the intrinsic nutritional value of these matrices, allowing their incorporation into food systems as cost‐effective sources of dietary fiber, proteins, and antioxidants. Therefore, the choice between extracted fractions and use of crude by‐products depends on the desired functionality, level of processing, and final application, with both approaches playing a crucial role in the development of functional and sustainable foods.

As summarized in Table 3, RB and its derivatives have been extensively used in bakery and cereal‐based products, primarily due to their high fiber and protein content. The incorporation of RB powder into biscuits at levels ranging from 5% to 30%, with an optimal substitution around 15%, has been shown to significantly enhance nutritional quality in terms of protein, ash, and fiber content, while maintaining acceptable sensory properties (Ayoub et al. 2022). Similarly, DRB, obtained after lipid extraction, has been successfully incorporated into bread at 18% substitution level, leading to a threefold increase in dietary fiber and additional health benefits, including improved lipid profile and modulation of gut microbiota (Ng et al. 2025). These findings highlight how even minimally processed by‐products can deliver both technological and physiological benefits.

**TABLE 3:** Rice by‐product applications as ingredient in food industry.

Rice by‐product	Extracted/Valorized compounds	Target/Scope	Processing method and conditions	References
Rice bran (RB)	Fiber and protein	Enriched biscuit	RB powder (5%–30%) substituted in wheat flour, alone or with wheat bran/banana peel. Optimal at 15% RB: increased protein, ash, fiber; reduced diameter, higher spread ratio; acceptable sensory quality	Ayoub et al. (2022)
Defatted rice bran (DRB)	Dietary fiber (DF)	Bread	Defatted rice bran was incorporated into white bread at 18% flour replacement and tested in a 4‐week randomized crossover trial (3–4 slices/day). The bread tripled dietary fiber compared with the control, increased HDL cholesterol, lowered the TC/HDL ratio, and modulated gut microbiota by increasing *Faecalibacterium prausnitzii*	Ng et al. (2025)
	High dietary fiber and shelf‐life improver	Chicken sausage	Defatted rice bran incorporated at 5%–15% increased fiber, ash, and antioxidant stability. The best sensory acceptability was observed at 5%, with extended shelf life reflected by lower FFA and TBARS compared with the control	Sagar et al. (2024)
	Soluble dietary fiber	Fiber‐enriched with reduced GI biscuit	Soluble dietary fiber (F‐SDF) was extracted from defatted rice bran using *Trichoderma viride* fermentation, enzymatic hydrolysis (α‐amylase, papain), ethanol precipitation, and freeze‐drying. When incorporated into biscuits (1.2%–10.8%), it reduced hardness, disrupted the gluten network, lowered predicted GI, and showed highest acceptability at 6% addition	Jia et al. (2020)
Rice bran protein (DRBP)	Defatted rice bran protein (DRBP)	Fruit‐juice beverage fortified with RB protein	Defatted rice bran was hydrolyzed with Alcalase (0.01%, 50°C, 30 min, pH 8), inactivated at 85°C for 10 min then treated with Flavourzyme (0.075%, 50°C, 4 h, pH 6). The hydrolysate was freeze‐dried, and incorporated at 2% into juice, followed by pasteurization at 75°C for 2 min 50 s	Saveboworn et al. (2025)
	Rice bran protein (RBP)	Foamable protein ingredient for aerated foods: meringue/ice cream/bakery foams	Rice bran was defatted with *n*‐hexane (1:5, 55°C, 1 h ×5), alkali‐extracted (pH 9, 50°C, 2 h), centrifuged (approximately 9986 × *g*), precipitated at pH 4.5 (4°C, overnight), washed, and freeze‐dried to obtain RBP (NSI > 90%). TGase cross‐linking of 30 mg/mL RBP at 40°C (0.3–0.9 kat/kg, 2–5 h) showed optimal performance at 0.5 kat/kg for 3 h, yielding foam capacity approximately 375%, foam‐volume ratio approximately 79.5%, and enhanced interfacial dilatational modulus, indicating Pickering‐type stabilization	Wu et al. (2023)
Stabilized rice bran (SRB)	Antioxidant and dietary fiber	Bread	Stabilized rice bran (autoclaved at 120°C, 5 psi, 20 min; air‐dried at 70°C, 20 min; sieved 500 µm) was incorporated into wheat bread at 10%–25%	Espinales et al. (2022)
	High dietary fiber, fat, antioxidants	Hybrid chicken patties	Stabilized rice bran autoclaved at 121°C for 20 min was incorporated at 0%–8% replacing chicken meat in patties formulated with 30% pumpkin and carrot	Aviles et al. (2023)
	High γ‐oryzanol, phytochemicals, dietary fiber	Functional food ingredients	Rice bran stabilized by dry heat or twin‐screw extrusion (120°C, 130 rpm, 21% moisture) was stored for 60 days at 15°C, 25°C, and 40°C	Rashid et al. (2023)
	Functional ingredient	Infant foods and protein‐enriched products	Dry‐heat treatment of rice bran at 120°C for 233–88 min or 130°C for 86–50 min effectively inactivated enzymes and improved storage stability	Rashid et al. (2022)
Rice bran oil (RBO)	Nanofibers	Emollients, cosmetic nanostructures	Electrospinning of rice bran oil with ethyl cellulose/PVP in ethanol produced nanofibers with high encapsulation efficiency, stability, and nontoxicity, suitable for cosmetic and cosmeceutical applications	Lima et al. (2023)
	Coating emulsion	Edible egg‐coating emulsion as a preservative	Edible coatings were prepared with gellan gum (0.10–0.20% w/v) with or without basil essential oil (0.50% v/v) and glycerol, emulsified with 20% rice bran oil, and applied to eggs pre‐washed in 1% sodium percarbonate	Pan et al. (2023)
	Rice bran wax oleogel	Oxidative stability	Crude wax obtained during rice bran oil refining was crystallized into oleogels containing 7–11 wt% wax, which were stable at 20°C and showed improved oxidative stability	Wang et al. (2021)
Rice bran oil (RBO) and rice bran wax	Oleogels	Fat replacer in Thai sweet sausage (Goon Chiang)	Rice bran wax (2% w/w) with rice bran oil was heated to 80°C, cooled, and left overnight to form an oleogel (RBOG). When incorporated into sausages, RBOG improved texture, reduced SFA and cholesterol, increased USFA, and enhanced sensory acceptance compared with pork fat	Issara (2022)
Rice bran wax	Oleogels	Shortening replacer in cookies	Oleogels were prepared with 3–9 wt% rice bran wax in expeller‐pressed or refined corn germ oil by heating at 120°C, cooling to room temperature, and storing at 5°C for 7 days. When tested in cookies as shortening alternatives, the oleogels showed functional applicability	Zhao et al. (2020)
Rice bran lecithin (RBL)	Nanoemulsions	Natural emulsifier	De‐oiled rice bran lecithin (1%–5% w/w) combined with xanthan gum was used as a natural emulsifier in soybean oil‐in‐water emulsions. Ultrasonication (20 kHz, 60% amplitude, 40 cycles) produced nanoemulsions (10–150 nm) with high kinetic stability, negative zeta potential, and low creaming index	Lehri et al. (2021)
Rice husk flour (RHF)	High dietary fiber	Fortified beef sausages	Rice husk flour was prepared by washing (50°C, 15 min), oven drying (60°C), milling, and sieving (0.20 mm), and incorporated into sausages at 0%–15% replacing wheat flour. Cooking at 200°C for 20 min, RHF increased dietary fiber (up to 70%), reduced TBARS and 7‐ketocholesterol, and improved taste and juiciness, though cook loss increased at ≥10% RHF	Adeyemi et al. (2025)
Broken rice	Starch	Extruded (puffed) snack	Twin‐screw extrusion of broken rice‐cowpea blends (70–90:30–10) was optimized at a ratio of 85:15 with 12% moisture, 140°C barrel temperature, and 300 rpm screw speed, giving the best expansion and sensory acceptability	Okunola et al. (2023)
	Starch	Extruded snack with turmeric	Broken rice blended with 0%–10% turmeric was conditioned to 12.5% moisture for 24 h at 5°C, then processed by single‐screw extrusion (barrel 41/61/84°C, 4 mm die, 3:1 compression, approximately 335 g/min feed) followed by hot‐air drying at 100°C for 20 min	Ribeiro Oliveira et al. (2020)
	Liquid glucose	Ice cream	Broken rice slurry was hydrolyzed with α‐amylase and glucoamylase for liquefaction and saccharification, achieving DE 40–56, efficiency 25%–33%, and water activity 0.60–0.75. The product was tested in ice cream and showed high sensory acceptance (94.6%)	Sindhu et al. (2024)
Rice flour	Low‐protein rice drink	PKU patients drink	A formulation with 3% rice flour and 0.05% xanthan gum was heated at 80°C for 15 min and fortified with caseinomacropeptide concentrate. Sensory quality was further improved with 2.5% sucrose	Karimidastjerd and Kilic‐Akyilmaz (2021)
Rice starch	Fat replacer	Whipping cream	Raw and modified starches (retrograded, retrograded‐annealed) from three rice varieties with amylose contents of 22.7%, 9.8%, and 0.3% were used as fat replacers in cream (approximately 15% fat). They improved foam stability and overrun (44%) while reducing fat by 62% compared with the control	Iftikhar and Dutta (2020)

Protein‐rich fractions derived from RB, such as DRB protein (DRBP) and RBP isolates, are increasingly used as functional ingredients following extraction and enzymatic modification. As an example, enzymatically hydrolyzed DRBP has been incorporated into fruit juice beverages (2% addition), enabling protein fortification without compromising sensory quality (Saveboworn et al. 2025). In addition, extracted and structurally modified RBP exhibits excellent techno‐functional properties, including foaming and emulsifying capacity, allowing its application in aerated systems such as meringues, ice cream, and bakery foams (Wu et al. 2023). These applications demonstrate the importance of extraction and downstream modification in tailoring ingredient functionality for specific food systems.

Dietary fiber fractions, whether used as part of the whole matrix or extracted in soluble form, also play a key role in food applications. DRB incorporated into meat products (5%–15%) enhances fiber content and oxidative stability, contributing to extended shelf life and improved nutritional profile, with optimal sensory acceptance at lower inclusion levels (Sagar et al. 2024). Similarly, extracted SDF fractions, obtained through enzymatic processes, have been successfully used in biscuits to reduce hardness, lower predicted glycemic index, and improve overall acceptability, with optimal inclusion levels around 6% (Jia et al. 2020). RB, after the stabilization, obtained by the inactivation of lipase enzyme through thermal treatments such as autoclaving or extrusion, has also been widely used in bakery and meat products, typically at levels of 10%–25% in bread and up to 8% in meat formulations, contributing to improved nutritional and functional properties (Espinales et al. 2022; Aviles et al. 2023).

Furthermore, RBO and RB wax have been increasingly used in structured lipid systems such as oleogels, serving as fat replacers in meat products and bakery items and improving lipid profiles by reducing saturated fat content while maintaining desirable texture and sensory attributes (Issara 2022; Zhao et al. 2020). Additionally, RB lecithin extract has been employed as a natural emulsifier in nanoemulsion systems, enhancing stability and enabling its use in functional and formulated food products (Lehri et al. 2021). Beyond food applications, RBO has also been explored in cosmetic formulations, such as nanofibers and coating emulsions, highlighting its versatility (Lima et al. 2023; Pan et al. 2023).

Other rice by‐products, including RH flour and broken rice, also demonstrate significant application potential. RH flour, used as a fiber‐rich ingredient in meat products (0%–15%), has been shown to increase dietary fiber content and improve oxidative stability, although higher inclusion levels may affect technological properties such as cooking loss (Adeyemi et al. 2025). Broken rice, on the other hand, is widely utilized in starch‐based applications, including extruded snacks and glucose syrups, as well as in ice cream formulations, where enzymatically derived glucose syrups have demonstrated high sensory acceptance (Sindhu et al. 2024). Furthermore, rice starch and flour are commonly employed as fat replacers in specialized formulations, such as low‐protein beverages for clinical nutrition, contributing to improved texture and tailored nutritional profiles (Iftikhar and Dutta 2020; Karimidastjerd and Kilic‐Akyilmaz 2021).

Overall, the applications reported highlight a dual strategy in the valorization of rice by‐products: the direct use of whole or minimally processed materials for nutritional enrichment and the use of extracted and modified fractions for targeted functional performance. This combined approach maximizes the value of rice processing residues, enabling their transformation into versatile ingredients for a wide range of food and nonfood applications while supporting sustainability and circular economy principles.

## 6 Future Perspectives

The valorization of RB and its associated fractions offers a transformative opportunity to convert abundant agro‐industrial residues into a portfolio of high‐value functional ingredients and bio‐based products. Unlocking this potential depends on effective pretreatment and extraction. Conventional solvent‐based methods, like Soxhlet extraction, provide historical benchmarks but suffer from inefficiencies, solvent intensity, and limited selectivity (Gil‐Martín et al. 2022; Kumar et al. 2023). In contrast, intensified green technologies, including PLE, SWE, SFE, MAE, UAE, CP, PEF, OHAE, and EAE, enable higher yields, shorter times, lower solvent consumption, and better preservation of thermolabile compounds (Mehta et al. 2022; Xi et al. 2024). Hybrid and integrated systems that combine complementary mechanisms, such as UAE‐SFE or PEF pretreatment with EAE, demonstrate superior recovery and selectivity (Modupalli et al. 2024; Soares et al. 2018). Developing decision trees that match compound polarity, matrix recalcitrance, and heat sensitivity with extraction strategies will shift current practice from empirical optimization to rational process design (Carpentieri et al. 2021). As an example, defined strategies must be considered in choosing the right extraction technologies between pigmented and nonpigmented rice by‐products due to their distinct chemical composition and stability. Pigmented rice by‐products (e.g., black and red RB) are particularly rich in hydrophilic compounds such as anthocyanins, flavonoids, and bound phenolics, which are highly sensitive to temperature, pH, and oxidative conditions. Therefore, nonthermal and mild green extraction techniques, such as UAE, PEF, and CP, are more suitable for preserving these thermolabile compounds while enhancing their release through cell disruption and improved mass transfer (Chemat et al. 2017). In contrast, nonpigmented rice by‐products are dominated by lipophilic bioactives such as γ‐oryzanol, tocopherols, and fatty acids, which are more stable and better recovered using green solvent‐based or pressurized techniques, such as SFE and PLE (Herrero et al. 2010). Furthermore, the presence of both free and bound phenolic fractions in pigmented rice increases matrix complexity, requiring more advanced or combined extraction approaches, whereas nonpigmented matrices are generally less complex but more susceptible to lipid oxidation during processing and storage (Li et al. 2023). These differences highlight that the selection of green extraction technologies should be tailored according to the rice type: nonthermal, low‐intensity processes are preferred for pigmented rice to preserve sensitive metabolites, whereas high‐efficiency extraction systems targeting lipophilic compounds are more suitable for nonpigmented rice by‐products.

Furthermore, despite promising laboratory‐scale outcomes, several barriers impede industrial adoption. Equipment durability, electrode corrosion, uneven treatment in viscous matrices, and high CAPEX/OPEX remain critical challenges for OHAE, SWE, and SFE (Buljeta et al. 2023; Lee et al. 2025). Advances in reactor design, such as multi‐pin CP arrays or continuous‐flow microwave/ultrasound systems, highlight strategies to enhance uniformity and scalability (Fraterrigo Garofalo et al. 2021; Murakonda and Dwivedi 2024). Computational fluid dynamics (CFD), coupled with process analytical technologies, can further ensure reproducibility and energy efficiency at scale (Ali et al. 2026). EAE, while eco‐efficient, faces limitations from cost, incomplete hydrolysis, and emulsion formation. However, enzyme cocktails have delivered recovery rates above 90% for RBO while simultaneously releasing phenolics (Tufail et al. 2024). Progress in recombinant, immobilized, and engineered enzymes, along with pretreatments such as extrusion that restructure cell walls, can overcome current bottlenecks and enable continuous operation (Brondani Teixeira Ribas et al. 2025; Modupalli et al. 2024).

Sustainability claims around green extraction must be validated through systematic TEA and LCA. Although SFE is often promoted as solvent‐free, its high energy demand can offset environmental benefits (Chemat et al. 2020; Colombo et al. 2024). Similarly, SWE minimizes solvent footprints but requires energy audits and corrosion‐resistant equipment to ensure viability (Moreira et al. 2023). Early integration of TEA/LCA into research pipelines is essential for fair benchmarking and industrial acceptance (Usman et al. 2023).

On the bioactivity front, while antioxidant, hypocholesterolemic, and antihypertensive effects of rice‐derived fractions are well‐documented, most evidence remains at the in vitro or animal study stage. Clinical validation, including standardized digestion models, stability assays, and well‐powered human trials, is critical to support regulatory approval and consumer trust (Nidhishree et al. 2024; Peanparkdee and Iwamoto 2019). Prioritizing fractions such as γ‐oryzanol‐rich oils, protein hydrolysates, and XOS, combined with omics‐based biomarkers and microbiome endpoints, can accelerate translation to functional foods and nutraceuticals (Colla et al. 2025).

Finally, collaborative infrastructures will be vital to accelerate progress. Round‐robin studies on reference rice by‐products can establish reproducibility across laboratories, whereas open‐access datasets linking process conditions to yields and quality outcomes would enable data‐driven modeling and AI‐assisted optimization (Ran et al. 2019; Xi et al. 2024). Precompetitive consortia and shared pilot plants can reduce investment risk and harmonize protocols for green extraction under industrially relevant conditions (Buljeta et al. 2023; Carpentieri et al. 2021; Ran et al. 2019; Xi et al. 2024). Moreover, it is also important that the valorization of rice by‐products such as RS, RB, and RH can be significantly enhanced through the adoption of a biorefinery approach, which aligns with the principles of a circular economy. In a biorefinery framework, these agricultural residues are not treated as waste but as raw materials to produce multiple value‐added products, including biofuels, biochemicals, functional food ingredients, and biomaterials. For instance, RS can be fractionated to recover cellulose, hemicellulose, lignin, and bioactive compounds, whereas RB provides lipids, proteins, and antioxidants for nutraceutical and industrial applications (Martínez‐Guillén et al. 2025; Kamboj et al. 2024). Integrating rice by‐products into multiproduct biorefineries offers several advantages: It maximizes resource efficiency, reduces environmental impact, and generates income for farmers and industries. Furthermore, coupling biorefinery operations with green extraction technologies—such as EAE, supercritical fluid extraction, ultrasound, and microwave‐assisted methods—enhances process sustainability by reducing energy consumption and solvent use (Nadar et al. 2018; Mandal et al. 2007; Herrero et al. 2010).

From a circular economy perspective, this strategy promotes resource recirculation and minimizes waste generation, contributing to climate mitigation by reducing greenhouse gas emissions (Alexandri et al. 2020). However, the industrial implementation of rice biorefineries faces challenges, including high capital investment, optimization of multistep processing, variability of feedstock composition, and regulatory barriers.

Overall, the biorefinery concept represents a promising pathway to transform rice by‐products into sustainable sources of energy, chemicals, and functional ingredients, thus supporting the global transition toward a circular bioeconomy.

## 7 Conclusion

Rice by‐products, including RB, DRB, broken rice, husk, and straw, represent a highly diverse and underutilized resource with significant potential for the recovery of lipids, proteins, dietary fibers, phenolic compounds, phytosterols, tocopherols, γ‐oryzanol, anthocyanins, and mineral‐rich fractions. Unlike many other plant‐based residues, rice processing side streams combine nutritional richness with marked compositional heterogeneity, making them particularly attractive but also technically challenging matrices for valorization. In particular, the rapid enzymatic deterioration of RB, the recalcitrant lignocellulosic structure of husk and straw, and the coexistence of both hydrophilic and lipophilic bioactives require extraction strategies that are not only efficient but also selective and matrix‐specific.

The evidence reviewed in this work highlights that no single extraction technology is universally optimal for all rice by‐products. Instead, the most suitable approach depends on the target fraction, matrix structure, thermal sensitivity, and intended end use. Conventional methods remain useful as reference approaches and, in some cases, as complementary pretreatment or recovery steps, but their limitations in terms of solvent use, extraction time, and compound preservation are evident. In contrast, green and intensified technologies, such as SFE, PLE, SWE, UAE and MAE, PEFs, and EAE, offer more promising routes for the selective recovery of high‐value compounds from rice matrices. Their relevance is particularly evident in the extraction of lipophilic antioxidants from RB, phenolic compounds from pigmented rice fractions, and structural polysaccharides and fibers from husk and straw.

Overall, the valorization of rice by‐products should move beyond single‐compound recovery toward integrated cascade biorefinery models in which multiple fractions are sequentially recovered and directed toward food, nutraceutical, cosmetic, packaging, and bio‐based applications. Future progress in this field will depend on improved stabilization strategies, better standardization of extraction conditions, stronger techno‐economic and LCA, and clearer alignment between fraction characteristics and industrial application requirements. If these challenges are addressed, rice by‐products can evolve from low‐value residues into key raw materials for the development of sustainable, circular, and health‐oriented value chains.

## Author Contributions

**Shabnam Haghighat Khajavi**: conceptualization, investigation, methodology, writing – original draft. **Aidin Taromsari**: conceptualization, investigation, writing – original draft. **Kiana Moradi**: conceptualization, investigation, writing – original draft. **Maria Concetta Tenuta**: investigation, writing – review and editing. **Giovanna Ferrentino**: conceptualization, methodology, supervision, writing – review and editing.

## Conflicts of Interest

The authors declare no conflicts of interest.

## References

[crf370511-bib-0001] Abd Razak, D. L. , A. Jamaluddin , N. Y. Abd Rashid , A. Abd Ghani , and M. Abdul Manan . 2019. “Assessment of Fermented Broken Rice Extracts for Their Potential as Functional Ingredients in Cosmeceutical Products.” Annals of Agricultural Sciences 64, no. 2: 176–182. 10.1016/j.aoas.2019.11.003.

[crf370511-bib-0002] Adeyemi, K. D. , I. O. Kolade , A. O. Siyanbola , et al. 2025. “Rice Husk‐Fortified Beef Sausages: Cholesterol Oxidation Products, Physicochemical Properties, and Sensory Attributes.” Meat Science 220: 109714. 10.1016/j.meatsci.2024.109714. 39603142

[crf370511-bib-0003] Alexandri, M. , J. P. López‐Gómez , A. Olszewska‐Widdrat , and J. Venus . 2020. “Valorising Agro‐Industrial Wastes Within the Circular Bioeconomy Concept: The Case of Defatted Rice Bran With Emphasis on Bioconversion Strategies.” Fermentation 6, no. 2: 42. 10.3390/fermentation6020042.

[crf370511-bib-0004] Ali, M. A. , S. C. Chew , K. Lin Nyam , and K. Aryusuk . 2026. “Green Extraction Technologies, Antioxidant Mechanism and Therapeutic Potentials of Gamma‐Oryzanol.” Food Reviews International 42: 1587–1623. 10.1080/87559129.2025.2521362.

[crf370511-bib-0005] Aluthge, D. S. U. , K. K. D. S. Ranaweera , and I. A. D. S. R. Gunathilake . 2023. “The Effect of Stabilization Heat Treatment on Rice Bran Quality Parameters, Including Total Phenolic Content, Gamma Oryzanol Content, Antioxidant Potential, Oxidative Stability and Extraction Yield During Storage.” Food Chemistry Advances 3: 100531. 10.1016/j.focha.2023.100531.

[crf370511-bib-0172] Aluthge, S. , S. Gunathilake , C. Brennan , A. Farahnaky , and M. Majzoobi . 2025. “Conventional and Emerging Methods for Cereal By‐Product Valorisation.” Journal of Cereal Science 126: 104289. 10.1016/j.jcs.2025.104289.

[crf370511-bib-0006] Anyiam, P. N. , S. Phongthai , P. Kingwascharapong , et al. 2025. “A Comparative Review on Novel‐Assisted Extraction Techniques for Retrieving Protein From Some Potential Plant Sources.” NFS Journal 40: 100239. 10.1016/j.nfs.2025.100239.

[crf370511-bib-0007] Arellano, C. A. , A. Corpuz , and L. T. Nguyen . 2022. “Microwave‐Assisted Extraction for the Valorization of Agro‐Industrial Waste.” In Valorization of Agro‐Industrial Byproducts. CRC Press. 10.1201/9781003125679-3.

[crf370511-bib-0008] Athanasiadis, V. , T. Chatzimitakos , K. Kotsou , D. Kalompatsios , E. Bozinou , and S. I. Lalas . 2023. “Polyphenol Extraction From Food (by) Products by Pulsed Electric Field: A Review.” International Journal of Molecular Sciences 24, no. 21: 15914. 10.3390/ijms24211591. 37958898 PMC10650265

[crf370511-bib-0009] Aviles, M. V. , E. F. Naef , R. A. Abalos , L. H. Lound , M. B. Gómez , and D. F. Olivera . 2023. “Use of a Rice Industry By‐Product as a Meat Replacer in a Hybrid Chicken Patty: Technological and Sensory Impact.” International Journal of Gastronomy and Food Science 31: 100674. 10.1016/j.ijgfs.2023.100674.

[crf370511-bib-0010] Ayoub, W. S. , I. Zahoor , A. H. Dar , et al. 2022. “Effect of Incorporation of Wheat Bran, Rice Bran and Banana Peel Powder on the Mesostructure and Physicochemical Characteristics of Biscuits.” Frontiers in Nutrition 9: 1016717. 10.3389/fnut.2022.1016717. 36466403 PMC9714488

[crf370511-bib-0011] Babini, E. , D. L. Taneyo‐Saa , A. Tassoni , et al. 2020. “Microbial Fermentation of Industrial Rice‐Starch Byproduct as Valuable Source of Peptide Fractions With Health‐Related Activity.” Microorganisms 8, no. 7: 986. 10.3390/microorganisms8070986. 32630107 PMC7409224

[crf370511-bib-0012] Baixinho, J. P. , M. Cardeira , A. Bento‐Silva , et al. 2025. “Optimization of Supercritical Fluid Extraction for the Recovery of γ‐Oryzanol‐Rich Extracts With Improved Bioactivity From Rice Bran.” Antioxidants 14, no. 2: 206. 10.3390/antiox14020206. 40002392 PMC11852124

[crf370511-bib-0013] Balegh, S. M. , P. Saadati , M. Safiaghdam , A. A. Fakhari , and R. Razavi . 2025. “Effect of Cold Atmospheric Plasma Pre‐Treatment and Ultrasound‐Assisted Extraction on Bioactive Compounds and Chemical Quality of Rice Bran Oil.” LWT 218: 117451. 10.1016/j.lwt.2025.117451.

[crf370511-bib-0014] Barba, F. J. , O. Parniakov , S. A. Pereira , et al. 2015. “Current Applications and New Opportunities for the Use of Pulsed Electric Fields in Food Science and Industry.” Food Research International 77: 773–798. 10.1016/j.foodres.2015.09.015.

[crf370511-bib-0015] Begum, A. , J. Sarma , P. Borah , et al. 2015. “Microwave (MW) Energy in Enzyme Deactivation: Stabilization of Rice Bran From Few Widely Consumed Indigenous Rice Cultivars (*Oryza sativa* L.) From Eastern Himalayan Range.” Current Nutrition & Food Science 11: 240–245. 10.2174/1573401311666150521233113.

[crf370511-bib-0016] Benito‐Román, O. , S. Varona , M. T. Sanz , and S. Beltrán . 2019. “Valorization of Rice Bran: Modified Supercritical CO_2_ Extraction of Bioactive Compounds.” Journal of Industrial and Engineering Chemistry 80: 273–282. 10.1016/j.jiec.2019.08.005.

[crf370511-bib-0017] Borrelli, G. M. , and D. B. M. Ficco . 2025. “Cereals and Cereal‐Based Foods: Nutritional, Phytochemical Characterization and Processing Technologies.” Foods 14, no. 7: 1234. 10.3390/foods14071234. 40238465 PMC11988532

[crf370511-bib-0018] Brites, P. , M. I. S. Aguiar , J. Gonçalves , P. Ferreira , and C. Nunes . 2024. “Sustainable Valorisation of Bioactive Molecules From Rice Husks Through Hydrothermal Extraction for Chitosan‐Based Bioplastic Production.” International Journal of Biological Macromolecules 271: 132489. 10.1016/j.ijbiomac.2024.132489. 38777004

[crf370511-bib-0019] Brondani Teixeira Ribas, F. , H. Gasparetto , and N. P. Gonçalves Salau . 2025. “Rice Bran Oil Valorization: A Comprehensive Review of Minor Compounds, Extraction, Advancements, and Prospects.” ACS Food Science & Technology 5, no. 3: 877–897. 10.1021/acsfoodscitech.4c00781.

[crf370511-bib-0020] Buljeta, I. , D. Šubarić , J. Babić , A. Pichler , J. Šimunović , and M. Kopjar . 2023. “Extraction of Dietary Fibers From Plant‐Based Industry Waste: A Comprehensive Review.” Applied Sciences 13, no. 16: 9309. 10.3390/app13169309.

[crf370511-bib-0021] Bunmusik, W. , P. Suttiarporn , T. Phankaew , P. Thitisut , and T. Seangwattana . 2023. “The Effects of Solvent–Based Ultrasonic–Assisted Extraction of Bioactive Compounds and Antioxidant Activities From Pigmented Rice Bran.” Materials Today: Proceedings 77: 1073–1078. 10.1016/j.matpr.2022.11.391.

[crf370511-bib-0022] Butsat, S. , N. Weerapreeyakul , and S. Siriamornpun . 2009. “Changes in Phenolic Acids and Antioxidant Activity in Thai Rice Husk at Five Growth Stages During Grain Development.” Journal of Agricultural and Food Chemistry 57, no. 11: 4566–4571. 10.1021/jf9000549. 19432451

[crf370511-bib-0023] Carpentieri, S. , F. Soltanipour , G. Ferrari , G. Pataro , and F. Donsì . 2021. “Emerging Green Techniques for the Extraction of Antioxidants From Agri‐Food By‐Products as Promising Ingredients for the Food Industry.” Antioxidants 10, no. 9: 1417. 10.3390/antiox10091417. 34573049 PMC8471374

[crf370511-bib-0024] Chatzimitakos, T. , V. Athanasiadis , D. Kalompatsios , M. Mantiniotou , E. Bozinou , and S. I. Lalas . 2023. “Pulsed Electric Field Applications for the Extraction of Bioactive Compounds From Food Waste and By‐Products: A Critical Review.” Biomass 3, no. 4: 367–401. 10.3390/biomass3040022.

[crf370511-bib-0025] Cheetangdee, N. 2019. “Rice Phenolics: Extraction, Characterization, and Utilization in Foods.” In Polyphenols in Plants. Elsevier. 10.1016/B978-0-12-813768-0.00015-3.

[crf370511-bib-0026] Chemat, F. , M. Abert Vian , A.‐S. Fabiano‐Tixier , et al. 2020. “A Review of Sustainable and Intensified Techniques for Extraction of Food and Natural Products.” Green Chemistry 22, no. 8: 2325–2353. 10.1039/C9GC03878G.

[crf370511-bib-0027] Chemat, F. , N. Rombaut , A. G. Sicaire , A. Meullemiestre , A. S. Fabiano‐Tixier , and M. Abert‐Vian . 2017. “Ultrasound Assisted Extraction of Food and Natural Products.” Ultrasonics Sonochemistry 34: 540–560. 10.1016/j.ultsonch.2016.06.035. 27773280

[crf370511-bib-0028] Chen, Y. , Y. Ma , L. Dong , et al. 2019. “Extrusion and Fungal Fermentation Change the Profile and Antioxidant Activity of Free and Bound Phenolics in Rice Bran Together With the Phenolic Bioaccessibility.” LWT 115: 108461. 10.1016/j.lwt.2019.108461.

[crf370511-bib-0029] Colla, E. , C. Canan , M. P. Corso , D. A. Drunkler , R. A. Silva‐Buzanello , and L. L. Antunes . 2025. “Rice Bran Valorization: Use as a Source of Proteins, Carbohydrates, Oil, and Bioactive Compounds.” In Sustainable Management of Agro‐Food Waste. Elsevier. 10.1016/B978-0-443-23679-2.00003-3.

[crf370511-bib-0030] Colombo, R. , G. Moretto , M. Barberis , I. Frosi , and A. Papetti . 2024. “Rice Byproduct Compounds: From Green Extraction to Antioxidant Properties.” Antioxidants 13, no. 1: 35. 10.3390/antiox13010035. PMC10812773 38247461

[crf370511-bib-0031] Cravotto, G. , A. Binello , G. Merizzi , and M. Avogadro . 2004. “Improving Solvent‐Free Extraction of Policosanol From Rice Bran by High‐Intensity Ultrasound Treatment.” European Journal of Lipid Science & Technology 106: 147–151. 10.1002/ejlt.200300914.

[crf370511-bib-0032] Dafiqurrohman, H. , K. A. Safitri , M. I. B. Setyawan , A. Surjosatyo , and M. Aziz . 2022. “Gasification of Rice Wastes Toward Green and Sustainable Energy Production: A Review.” Journal of Cleaner Production 366: 132926. 10.1016/j.jclepro.2022.132926.

[crf370511-bib-0033] Dang, T. T. , and T. Vasanthan . 2019. “Modification of Rice Bran Dietary Fiber Concentrates Using Enzyme and Extrusion Cooking.” Food Hydrocolloids 89: 773–782. 10.1016/j.foodhyd.2018.11.024.

[crf370511-bib-0034] Das, P. P. , M. Z. Gul , A. M. Weber , et al. 2025. “Rice Bran Extraction and Stabilization Methods for Nutrient and Phytochemical Biofortification, Nutraceutical Development, and Dietary Supplementation.” Nutrition Reviews 83, no. 4: 692–712. 10.1093/nutrit/nuae174. 39657228 PMC11894254

[crf370511-bib-0035] Deng, G. F. , X. R. Xu , Y. Zhang , D. Li , R. Y. Gan , and H. B. Li . 2013. “Phenolic Compounds and Bioactivities of Pigmented Rice.” Critical Reviews in Food Science and Nutrition 53, no. 3: 296–306. 23216001 10.1080/10408398.2010.529624

[crf370511-bib-0036] Deng, Y. , W. Wang , S. Zhao , et al. 2022. “Ultrasound‐Assisted Extraction of Lipids as Food Components: Mechanism, Solvent, Feedstock, Quality Evaluation and Coupled Technologies—A Review.” Trends in Food Science & Technology 122: 83–96. 10.1016/j.tifs.2022.01.034.

[crf370511-bib-0037] Dias, M. I. , J. Pinela , T. C. S. P. Pires , et al. 2023. “Biotransformation of Rice Husk Into Phenolic Extracts by Combined Solid Fermentation and Enzymatic Treatment.” Biology and Life Sciences Forum 28: 12. 10.3390/blsf2023028012.

[crf370511-bib-0038] Echenique, J. V. F. , G. Alvarez‐Rivera , V. M. A. Luna , et al. 2024. “Pressurized Liquid Extraction With Ethanol in an Intermittent Process for Rice Bran Oil: Evaluation of Process Variables on the Content of β‐Sitosterol and Phenolic Compounds, Antioxidant Capacity, Acetylcholinesterase Inhibitory Activity, and Oil Quality.” LWT 207: 116650. 10.1016/j.lwt.2024.116650.

[crf370511-bib-0039] Espinales, C. , A. Cuesta , J. Tapia , et al. 2022. “The Effect of Stabilized Rice Bran Addition on Physicochemical, Sensory, and Techno‐Functional Properties of Bread.” Foods 11, no. 21: 3328. 10.3390/foods11213328. 36359940 PMC9656163

[crf370511-bib-0040] Fabian, C. , A. Ayucitra , S. Ismadji , and Y. H. Ju . 2011. “Isolation and Characterization of Starch From Defatted Rice Bran.” Journal of the Taiwan Institute of Chemical Engineers 42: 86–91. 10.1016/j.jtice.2010.03.013.

[crf370511-bib-0041] Fărcaș, A. C. , S. A. Socaci , S. A. Nemeș , et al. 2022. “An Update Regarding the Bioactive Compound of Cereal By‐Products: Health Benefits and Potential Applications.” Nutrients 14, no. 17: 3470. 10.3390/nu14173470. 36079730 PMC9460243

[crf370511-bib-0042] Fraterrigo Garofalo, S. , T. Tommasi , and D. Fino . 2021. “A Short Review of Green Extraction Technologies for Rice Bran Oil.” Biomass Conversion and Biorefinery 11, no. 2: 569–587. 10.1007/s13399-020-00846-3.

[crf370511-bib-0043] Freitas, P. A. V. , C. González‐Martínez , and A. Chiralt . 2020. “Application of Ultrasound Pre‐Treatment for Enhancing Extraction of Bioactive Compounds From Rice Straw.” Foods 9, no. 11: 1657. 10.3390/foods9111657. 33198371 PMC7697156

[crf370511-bib-0044] Frosi, I. , D. Vallelonga , R. Colombo , C. Milanese , and A. Papetti . 2023. “Valorization of Rice Husk (*Oryza sativa* L.) as a Source of In Vitro Antiglycative and Antioxidant Agents.” Foods 12, no. 3: 529. 10.3390/foods12030529. 36766058 PMC9914668

[crf370511-bib-0173] Galanakis, C. M. 2022. “Sustainable Applications for the Valorization of Cereal Processing By‐Products.” Foods 11: 241. 10.3390/foods11020241. 35053973 PMC8775229

[crf370511-bib-0045] Gil‐Martín, E. , T. Forbes‐Hernández , A. Romero , D. Cianciosi , F. Giampieri , and M. Battino . 2022. “Influence of the Extraction Method on the Recovery of Bioactive Phenolic Compounds From Food Industry By‐Products.” Food Chemistry 378: 131918. 10.1016/j.foodchem.2021.131918. 35085901

[crf370511-bib-0046] Guazzotti, S. , C. Pagliano , F. Dondero , and M. Manfredi . 2023. “Lipidomic Profiling of Rice Bran After Green Solid–Liquid Extractions for the Development of Circular Economy Approaches.” Foods 12, no. 2: 384. 10.3390/foods12020384. 36673474 PMC9857567

[crf370511-bib-0047] Guerrini, A. , I. Burlini , B. Huerta Lorenzo , et al. 2020. “Antioxidant and Antimicrobial Extracts Obtained From Agricultural By‐Products: Strategies for a Sustainable Recovery and Future Perspectives.” Food and Bioproducts Processing 124: 397–407. 10.1016/j.fbp.2020.10.003.

[crf370511-bib-0048] Hanmoungjai, P. , D. L. Pyle , and K. Niranjan . 2001. “Enzymatic Process for Extracting Oil and Protein From Rice Bran.” Journal of the American Oil Chemists Society 78: 817–821. 10.1007/s11746-001-0348-2.

[crf370511-bib-0049] Hanmoungjai, P. , D. L. Pyle , and K. Niranjan . 2002. “Enzyme‐Assisted Water‐Extraction of Oil and Protein From Rice Bran.” Journal of Chemical Technology 77: 771–776. 10.1002/jctb.635.

[crf370511-bib-0050] Hernandez, N. , M. E. Rodriguez‐Alegria , F. Gonzalez , and A. Lopez Munguia . 2000. “Enzymatic Treatment of Rice Bran to Improve Processing.” Journal of the American Oil Chemists Society 77: 177–180. 10.1007/s11746-000-0028-2.

[crf370511-bib-0051] Herrero, M. , A. Cifuentes , and E. Ibáñez . 2010. “Sub‐ and Supercritical Fluid Extraction of Functional Ingredients.” Food Chemistry 98, no. 1: 136–148. 10.1016/j.foodchem.2005.05.058.

[crf370511-bib-0052] Huang, W. , B. Liu , D. Shi , et al. 2024. “Research Progress on the Quality, Extraction Technology, Food Application, and Physiological Function of Rice Bran Oil.” Foods 13, no. 20: 3262. 10.3390/foods13203262. 39456324 PMC11507353

[crf370511-bib-0053] Iftikhar, S. A. , and H. Dutta . 2020. “Use of Raw and Physically Modified Rice Starches as Fat Replacer in Whipping Cream.” Current Research in Nutrition and Food Science Journal 8, no. 1: 122–130. 10.12944/CRNFSJ.8.1.11.

[crf370511-bib-0054] Illankoon, W. A. M. A. N. , C. Milanese , M. C. Collivignarelli , and S. Sorlini . 2023. “Value Chain Analysis of Rice Industry by Products in a Circular Economy Context: A Review.” Waste 1, no. 2: 333–369. 10.3390/waste102002.

[crf370511-bib-0055] Irakli, M. , A. Lazaridou , and C. G. Biliaderis . 2020. “Comparative Evaluation of the Nutritional, Antinutritional, Functional, and Bioactivity Attributes of Rice Bran Stabilized by Different Heat Treatments.” Foods 10, no. 1: 57. 10.3390/foods10010057. 33379306 PMC7824238

[crf370511-bib-0056] Ishida, S. , S. Kudo , S. Asano , and J. Hayashi . 2025. “Multi‐Step Pre‐Treatment of Rice Husk for Fractionation of Components Including Silica.” Frontiers in Chemistry 13: 1538797. 10.3389/fchem.2025.1538797. 39917047 PMC11798958

[crf370511-bib-0057] Islam, M. , P. Saini , R. Das , S. Shekhar , A. S. K. Sinha , and K. Prasad . 2022. “Rice Straw as a Source of Nanocellulose for Sustainable Food Packaging Materials: A Review.” BioResources 18, no. 1: 2351–2385. 10.15376/biores.18.1.Islam.

[crf370511-bib-0058] Issara, U. 2022. “Improvement of Thai Sweet Sausage (Goon Chiang) Properties by Oleogel Made of Rice Bran Wax and Rice Bran Oil: A Textural, Sensorial, and Nutritional Aspect.” IOP Conference Series: Earth and Environmental Science 995, no. 1: 012045. 10.1088/1755-1315/995/1/012045.

[crf370511-bib-0059] Jafarzadeh, S. , Z. Qazanfarzadeh , E. Parandi , et al. 2025. “Eco‐Friendly Plastics From Cereal‐Derived By‐Products and Waste: A Circular Economy Approach for Sustainable Packaging.” Materials Today Chemistry 47: 102782. 10.1016/j.mtchem.2025.102782.

[crf370511-bib-0060] Jaichakan, P. , M. Nakphaichit , S. Rungchang , M. Weerawatanakorn , S. Phongthai , and W. Klangpetch . 2021. “Two‐Stage Processing for Xylooligosaccharide Recovery From Rice By‐Products and Evaluation of Products: Promotion of Lactic Acid‐Producing Bacterial Growth and Food Application in a High‐Pressure Process.” Food Research International 147: 110529. 10.1016/j.foodres.2021.110529. 34399507

[crf370511-bib-0061] Jha, P. , A. J. Das , and S. C. Deka . 2017. “Optimization of Ultrasound and Microwave Assisted Extractions of Polyphenols From Black Rice (*Oryza sativa* cv. Poireton) Husk.” Journal of Food Science and Technology 54, no. 12: 3847–3858. 10.1007/s13197-017-2832-0. 29085127 PMC5643800

[crf370511-bib-0062] Jia, M. , Q. Yu , J. Chen , et al. 2020. “Physical Quality and In Vitro Starch Digestibility of Biscuits as Affected by Addition of Soluble Dietary Fiber From Defatted Rice Bran.” Food Hydrocolloids 99: 105349. 10.1016/j.foodhyd.2019.105349.

[crf370511-bib-0063] Kamboj, A. , P. K. Sadh , P. Chawla , et al. 2024. “Sustainable Management of Rice By‐Products: Environmental Challenges, Industrial Applications, and Circular Bio‐Economy Innovations.” Biocatalysis and Agricultural Biotechnology 62: 103430. 10.1016/j.bcab.2024.103430.

[crf370511-bib-0064] Karimidastjerd, A. , and M. Kilic‐Akyilmaz . 2021. “Formulation of a Low‐Protein Rice Drink Fortified With Caseinomacropeptide Concentrate.” Food and Bioproducts Processing 125: 161–169. 10.1016/j.fbp.2020.11.004.

[crf370511-bib-0065] Kaur, S. , and A. Ubeyitogullari . 2023. “Extraction of Phenolic Compounds From Rice Husk via Ethanol‐Water‐Modified Supercritical Carbon Dioxide.” Heliyon 9, no. 3: e14196. 10.1016/j.heliyon.2023.e14196. 36938479 PMC10018476

[crf370511-bib-0066] Kayathi, A. , P. P. Chakrabarti , L. Bonfim‐Rocha , L. Cardozo‐Filho , A. Bollampalli , and V. Jegatheesan . 2021. “Extraction of γ‐Oryzanol From Defatted Rice Bran Using Supercritical Carbon Dioxide (SC‐CO_2_): Process Optimisation of Extract Yield, Scale‐Up and Economic Analysis.” Process Safety and Environmental Protection 148: 179–188. 10.1016/j.psep.2020.09.067.

[crf370511-bib-0067] KC, Y. , J. Mitchell , B. Bhandari , and S. Prakash . 2024. “Unlocking the Potential of Rice Bran Through Extrusion: A Systematic Review.” Sustainable Food Technology 2, no. 3: 594–614. 10.1039/D4FB00027G.

[crf370511-bib-0068] Khalid, S. , S. A. Hassan , A. B. Altemimi , et al. 2024. “Recovery of Valuable Substances From Food Waste by Ohmic Heating Assisted Extraction—A Step Towards Sustainable Production.” Future Foods 9: 100365. 10.1016/j.fufo.2024.100365.

[crf370511-bib-0069] Kovačević, D. B. , F. J. Barba , D. Granato , et al. 2018. “Pressurized Hot Water Extraction (PHWE) for the Green Recovery of Bioactive Compounds and Steviol Glycosides From *Stevia rebaudiana* Bertoni Leaves.” Food Chemistry 254: 150–157. 10.1016/j.foodchem.2018.01.192. 29548436

[crf370511-bib-0070] Koysuren, B. , M. H. Oztop , and B. G. Mazie . 2021. “Sesame Seed as an Alternative Plant Protein Source: A Comprehensive Physiochemical Characterization Study for Alkaline, Salt and Enzyme‐Assisted Extracted Samples.” International Journal of Food Science & Technology 56, no. 11: 5471–5484. 10.1111/ijfs.15229.

[crf370511-bib-0071] Krakowska‐Sieprawska, A. , A. Kiełbasa , K. Rafińska , M. Ligor , and B. Buszewski . 2022. “Modern Methods of Pre‐Treatment of Plant Material for the Extraction of Bioactive Compounds.” Molecules 27, no. 3: 730. 10.3390/molecules27030730. 35163995 PMC8840492

[crf370511-bib-0072] Krivošija, S. , A. Ballesteros‐Gómez , M. Zloh , et al. 2025. “Subcritical Water and Pressurised Ethanol Extractions for Maximum Recovery of Antioxidants From Orange Peel Herbal Dust With Evaluation of Its Pharmacological Potential Using In Silico and *In Vitro* Analysis.” Antioxidants 14, no. 6: 638. 10.3390/antiox14060638. 40563273 PMC12189749

[crf370511-bib-0073] Kumar, A. , N. P , M. Kumar , et al. 2023. “Major Phytochemicals: Recent Advances in Health Benefits and Extraction Method.” Molecules 28, no. 2: 887. 10.3390/molecules28020887. 36677944 PMC9862941

[crf370511-bib-0074] Kyu, M. T. , B. Dar , S. S. Aye , and T. Matsuda . 2019. “Prebiotic Oligosaccharides Prepared by Enzymatic Degradation of Dietary Fibers in Rice Grains.” Journal of Nutritional Science and Vitaminology 65, no. S: S143–S147. 10.3177/jnsv.65.S143. 31619616

[crf370511-bib-0075] Lavanya, M. N. , K. C. H. S. Saikiran , and N. Venkatachalapathy . 2019. “Stabilization of Rice Bran Milling Fractions Using Microwave Heating and Its Effect on Storage.” Journal of Food Science and Technology 56, no. 2: 889–895. 10.1007/s13197-018-3550-y. 30906046 PMC6400786

[crf370511-bib-0076] Lee, J. W. , J. Nam , W. Lim , T.‐G. Lim , and G.‐P. Hong . 2025. “Subcritical Water Mediated Hydrolysis of Rice Husk Lignocellulose and Upcycling Applications of the Hydrolysates.” Food and Bioprocess Technology 18, no. 1: 338–348. 10.1007/s11947-024-03477-y.

[crf370511-bib-0077] Lee, L. S. , N. Lee , Y. H. Kim , et al. 2013. “Optimization of Ultrasonic Extraction of Phenolic Antioxidants From Green Tea Using Response Surface Methodology.” Molecules 18, no. 11: 13530–13545. 10.3390/molecules181113530. 24184822 PMC6270505

[crf370511-bib-0078] Lehri, D. , N. Kumari , and R. P. Singh . 2021. “Ultrasound‐Assisted Production and Characterization of Rice Bran Lecithin‐Based Nanoemulsions.” Journal of Dispersion Science and Technology 42, no. 9: 1368–1375. 10.1080/01932691.2020.1764368.

[crf370511-bib-0079] Leonarski, E. , M. Kuasnei , P. A. D. Moraes , K. Cesca , D. de Oliveira , and A. A. F. Zielinski . 2023. “Pressurized Liquid Extraction as an Eco‐Friendly Approach to Recover Anthocyanin From Black Rice Bran.” Innovative Food Science & Emerging Technologies 86: 103372. 10.1016/j.ifset.2023.103372.

[crf370511-bib-0080] Li, H. , T. Liu , F. Li , X. Wu , and W. Wu . 2023. “Effects of Rice Bran Rancidity on Phenolics and Antioxidant Activity.” Food Research International 173: 113483. 37803806 10.1016/j.foodres.2023.113483

[crf370511-bib-0081] Lima, L. L. , K. Bispo‐dos‐Santos , I. M. C. Trevisan , et al. 2023. “Developing Botanical Formulations for Sustainable Cosmetics.” Cosmetics 10, no. 6: 159. 10.3390/cosmetics10060159.

[crf370511-bib-0082] Liu, L. , W. Wen , R. Zhang , et al. 2017. “Complex Enzyme Hydrolysis Releases Antioxidative Phenolics From Rice Bran.” Food Chemistry 214: 1–8. 10.1016/j.foodchem.2016.07.038. 27507440

[crf370511-bib-0083] Liu, Y. Q. , P. Strappe , Z. K. Zhou , and C. Blanchard . 2019. “Impact on the Nutritional Attributes of Rice Bran Following Various Stabilization Procedures.” Critical Reviews in Food Science and Nutrition 59, no. 15: 2458–2466. 10.1080/10408398.2018.1455638. 29561644

[crf370511-bib-0084] Ly, T. B. , N. T. T. Tran , C. D. Pham , D. D. B. Nguyen , P. T. Mai , and P. K. Le . 2024. “Innovative Method for Rice Straw Valorization Into Nanocellulose, Lignin and Silica.” Bioresource Technology Reports 25: 101804. 10.1016/j.biteb.2024.101804.

[crf370511-bib-0085] Mandal, V. , Y. Mohan , and S. Hemalatha . 2007. “Microwave Assisted Extraction—An Innovative and Promising Extraction Tool.” Pharmacognosy Reviews 1, no. 1: 7–18.

[crf370511-bib-0086] Manzoor, A. , V. K. Pandey , A. H. Dar , et al. 2023. “Rice Bran: Nutritional, Phytochemical, and Pharmacological Profile and Its Contribution to Human Health Promotion.” Food Chemistry Advances 2: 100296. 10.1016/j.focha.2023.100296.

[crf370511-bib-0087] Marathe, S. J. , S. B. Jadhav , S. B. Bankar , K. Kumari Dubey , and R. S. Singhal . 2019. “Improvements in the Extraction of Bioactive Compounds by Enzymes.” Current Opinion in Food Science 25: 62–72. 10.1016/j.cofs.2019.02.009.

[crf370511-bib-0088] Marín, M. , A. Sánchez , and A. Artola . 2019. “Production and Recovery of Cellulases Through Solid‐State Fermentation of Selected Lignocellulosic Wastes.” Journal of Cleaner Production 209: 937–946. 10.1016/j.jclepro.2018.10.264.

[crf370511-bib-0089] Martínez‐Guillén, J. R. , F. J. Álvarez‐Martínez , V. Micol , and E. Barrajón‐Catalán . 2025. “More Than a By‐Product: Scientific Advances on the Valorization of Rice Straw for a Greener Future.” Industrial Crops and Products 230: 121101. 10.1016/j.indcrop.2025.121101.

[crf370511-bib-0090] Mathias, M. G. , I. Okajima , K. Ito , Y. Aoki , C. Y. Kong , and T. Sako . 2023. “Influence of Extraction and Pretreatment Conditions on the Yield, Solubility, and Quality of Rice Bran Oil Extracted With CO_2_‐Expanded Hexane.” BioEnergy Research 16, no. 3: 1695–1705. 10.1007/s12155-022-10542-x.

[crf370511-bib-0091] Mehta, D. , K. Yadav , K. Chaturvedi , U. S. Shivhare , and S. K. Yadav . 2022. “Impact of Cold Plasma on Extraction of Polyphenol From De‐Oiled Rice and Corn Bran: Improvement in Extraction Efficiency, In Vitro Digestibility, Antioxidant Activity, Cytotoxicity and Anti‐Inflammatory Responses.” Food and Bioprocess Technology 15, no. 5: 1142–1156. 10.1007/s11947-022-02801-8.

[crf370511-bib-0092] Misnal, M. F. I. , N. Redzuan , M. N. F. Zainal , N. Ahmad , R. K. Raja Ibrahim , and L. Agun . 2022. “Cold Plasma: A Potential Alternative for Rice Grain Postharvest Treatment Management in Malaysia.” Rice Science 29, no. 1: 1–15. 10.1016/j.rsci.2021.12.001.

[crf370511-bib-0093] Modupalli, N. , A. Krisshnan , S. C K , et al. 2024. “Effect of Novel Combination Processing Technologies on Extraction and Quality of Rice Bran Oil.” Critical Reviews in Food Science and Nutrition 64, no. 7: 1911–1933. 10.1080/10408398.2022.2119367. 36106441

[crf370511-bib-0094] Mondal, P. , A. K. Sadhukhan , A. Ganguly , and P. Gupta . 2021. “Optimization of Process Parameters for Bio‐Enzymatic and Enzymatic Saccharification of Waste Broken Rice for Ethanol Production Using Response Surface Methodology and Artificial Neural Network–Genetic Algorithm.” 3 Biotech 11, no. 1: 28. 10.1007/s13205-020-02553-2. PMC7779392 33442526

[crf370511-bib-0095] Moreira, B. P. , C. P. Draszewski , N. C. Rosa , et al. 2023. “Integrated Rice Bran Processing by Supercritical CO_2_ Extraction and Subcritical Water Hydrolysis to Obtain Oil, Fermentable Sugars, and Platform Chemicals.” Journal of Supercritical Fluids 192: 105786. 10.1016/j.supflu.2022.105786.

[crf370511-bib-0096] Murakonda, S. , and M. Dwivedi . 2024. “Combined Use of Pulse Ultrasound–Assisted Extraction With Atmospheric Cold Plasma: Extraction and Characterization of Bioactive Compounds From Wood Apple Shell (*Limonia acidissima*).” Biomass Conversion and Biorefinery 14, no. 22: 28233–28251. 10.1007/s13399-022-03448-3.

[crf370511-bib-0097] Mustafa, A. , and C. Turner . 2011. “Pressurized Liquid Extraction as a Green Approach in Food and Herbal Plants Extraction.” Analytica Chimica Acta 703, no. 1: 8–18. 10.1016/j.aca.2011.07.018. 21843670

[crf370511-bib-0098] Nadar, S. S. , P. Rao , and V. K. Rathod . 2018. “Enzyme Assisted Extraction of Biomolecules as an Approach to Novel Extraction Technology: A Review.” Food Research International 108: 309–330. 10.1016/j.foodres.2018.03.006. 29735063

[crf370511-bib-0099] Ng, H. M. , J. Maggo , C. L. Wall , et al. 2025. “Effects of Defatted Rice Bran‐Fortified Bread on Gut Microbiome, Cardiovascular Risk, Gut Discomfort, Wellbeing and Gut Physiology in Healthy Adults With Low Dietary Fibre Intake.” Clinical Nutrition ESPEN 67: 362–376. 10.1016/j.clnesp.2025.03.045. 40127766

[crf370511-bib-0100] Nidhishree, A. S. , R. A. Menezes , H. Venkatachalam , and K. S. Bhat . 2024. “Rice Bran as a Sustainable Source for Value Added Materials: An Overview.” Discover Materials 4, no. 1: 93. 10.1007/s43939-024-00159-6.

[crf370511-bib-0101] Nzereogu, P. U. , A. D. Omah , F. I. Ezema , E. I. Iwuoha , and A. C. Nwanya . 2023. “Silica Extraction From Rice Husk: Comprehensive Review and Applications.” Hybrid Advances 4: 100111. 10.1016/j.hybadv.2023.100111.

[crf370511-bib-0102] Okunola, A. A. , E. P. Dottie , O. I. Moses , et al. 2023. “Development and Process Optimization of a Ready‐to‐Eat Snack From Rice‐Cowpea Composite by a Twin Extruder.” Processes 11, no. 7: 2159. 10.3390/pr11072159.

[crf370511-bib-0103] Pan, D. , Y. Li , Y. Hu , et al. 2023. “Effect of Different Concentrations of Gellan Gum With/Without 0.50% Basil Essential Oil on the Physicochemical Properties of Gellan Gum‐Rice Bran Oil Coating Emulsions and Their Application in Egg Preservation.” Food Chemistry 418: 136030. 10.1016/j.foodchem.2023.136030. 37004315

[crf370511-bib-0104] Pankaj, S. K. , N. N. Misra , K. J. Alzahrani , A. S. Alamri , and C. M. Galanakis . 2025. “Cold Plasma Treatment of Orange Juice Using Multipin‐Plane Electrical Discharge.” Journal of Food Process Engineering 48: e70072. 10.1111/jfpe.7007.

[crf370511-bib-0105] Peanparkdee, M. , and S. Iwamoto . 2019. “Bioactive Compounds From By‐Products of Rice Cultivation and Rice Processing: Extraction and Application in the Food and Pharmaceutical Industries.” Trends in Food Science & Technology 86: 109–117. 10.1016/j.tifs.2019.02.041.

[crf370511-bib-0106] Pinto, T. I. , J. A. Coelho , B. I. Pires , et al. 2021. “Supercritical Carbon Dioxide Extraction, Antioxidant Activity, and Fatty Acid Composition of Bran Oil From Rice Varieties Cultivated in Portugal.” Separations 8, no. 8: 115. 10.3390/separations8080115.

[crf370511-bib-0107] Punia, S. , M. Kumar , A. K. Siroha , and S. S. Purewal . 2021. “Rice Bran Oil: Emerging Trends in Extraction, Health Benefit, and Its Industrial Application.” Rice Science 28, no. 3: 217–232. 10.1016/j.rsci.2021.04.002.

[crf370511-bib-0108] Quagliariello, V. , R. V. Iaffaioli , M. Falcone , G. Ferrari , G. Pataro , and F. Donsì . 2016. “Effect of Pulsed Electric Fields—Assisted Extraction on Anti‐Inflammatory and Cytotoxic Activity of Brown Rice Bioactive Compounds.” Food Research International 87: 115–124. 10.1016/j.foodres.2016.07.005. 29606232

[crf370511-bib-0109] Ramos, M. , E. Laveriano , L. San Sebastián , et al. 2023. “Rice Straw as a Valuable Source of Cellulose and Polyphenols: Applications in the Food Industry.” Trends in Food Science & Technology 131: 14–27. 10.1016/j.tifs.2022.11.020.

[crf370511-bib-0110] Ramos, P. R. , J. Sponchiado , J. V. F. Echenique , G. C. Dacanal , and A. L. d. Oliveira . 2024. “Kinetics of Vegetable Oils (Rice Bran, Sunflower Seed, and Soybean) Extracted by Pressurized Liquid Extraction in Intermittent Process.” Processes 12, no. 6: 1107. 10.3390/pr12061107.

[crf370511-bib-0111] Rampelotto de Azevedo, A. , M. S. Nascimento dos Santos , C. Perinazzo Draszewski , et al. 2023. “Combined Ultrasonic/Subcritical Water Hydrolysis Pretreatments for Agricultural Biomass.” Environmental Technology 44, no. 19: 2969–2982. 10.1080/09593330.2022.2048088. 35226584

[crf370511-bib-0112] Ran, X. , M. Zhang , Y. Wang , and B. Adhikari . 2019. “Novel Technologies Applied for Recovery and Value Addition of High Value Compounds From Plant Byproducts: A Review.” Critical Reviews in Food Science and Nutrition 59, no. 3: 450–461. 10.1080/10408398.2017.1377149. 28920702

[crf370511-bib-0113] Rashid, M. T. , K. Liu , S. Han , and M. A. Jatoi . 2022. “The Effects of Thermal Treatment on Lipid Oxidation, Protein Changes, and Storage Stabilization of Rice Bran.” Foods 11, no. 24: 4001. 10.3390/foods11244001. 36553743 PMC9778295

[crf370511-bib-0114] Rashid, M. T. , K. Liu , S. Hen , M. A. Jatoi , and F. Sarpong . 2023. “Nutritional Composition and Volatile Compounds Stability in Dry‐Heat and Extruded Stabilised Rice Bran During Storage.” International Journal of Food Science & Technology 58, no. 6: 3379–3391. 10.1111/ijfs.16323.

[crf370511-bib-0115] Requena, R. , A. Jiménez‐Quero , M. Vargas , R. Moriana , A. Chiralt , and F. Vilaplana . 2019. “Integral Fractionation of Rice Husks Into Bioactive Arabinoxylans, Cellulose Nanocrystals, and Silica Particles.” ACS Sustainable Chemistry & Engineering 7, no. 6: 6275–6286. 10.1021/acssuschemeng.8b06692.

[crf370511-bib-0116] Ribas, F. B. T. , H. Gasparetto , and N. P. G. Salau . 2023. “Sustainable Extraction of Rice Bran Oil: Assessing Renewable Solvents, Kinetics, and Thermodynamics.” Chemical Engineering Research & Design 197: 342–354. 10.1016/j.cherd.2023.07.047.

[crf370511-bib-0117] Ribeiro Oliveira, A. , A. E. Chaves Ribeiro , É. Resende Oliveira , et al. 2020. “Physicochemical, Microbiological and Sensory Characteristics of Snacks Developed From Broken Rice Grains and Turmeric Powder.” International Journal of Food Science & Technology 55, no. 7: 2719–2729. 10.1111/ijfs.14525.

[crf370511-bib-0118] Ritthibut, N. , S.‐J. Oh , and S.‐T. Lim . 2021. “Enhancement of Bioactivity of Rice Bran by Solid‐State Fermentation With *Aspergillus* Strains.” LWT 135: 110273. 10.1016/j.lwt.2020.110273.

[crf370511-bib-0119] Rodríguez‐Restrepo, Y. A. , P. Ferreira‐Santos , C. E. Orrego , J. A. Teixeira , and C. M. R. Rocha . 2020. “Valorization of Rice By‐Products: Protein‐Phenolic Based Fractions With Bioactive Potential.” Journal of Cereal Science 95: 103039. 10.1016/j.jcs.2020.103039.

[crf370511-bib-0120] Roy, T. , A. Pawar , A. Singh , G. L. Loushigam , and M. D. Wagh . 2025. “A Comprehensive Review on Rice Proteins: Composition, Structural Modification, Functional and Industrial Food Applications.” Critical Reviews in Food Science and Nutrition 65: 8842–8859. 10.1080/10408398.2025.2511230. 40454583

[crf370511-bib-0121] Sagar, M. , M. Habib , M. Hashem , M. Azad , M. Rahman , and M. Ali . 2024. “Development of Dietary Fiber Enriched Sausage Using Rice Bran.” Meat Research 4, no. 2: 1–7. 10.55002/mr.4.2.87.

[crf370511-bib-0122] Sakr, M. , and S. Liu . 2014. “A Comprehensive Review on Applications of Ohmic Heating (OH).” Renewable and Sustainable Energy Reviews 39: 262–269. 10.1016/j.rser.2014.07.061.

[crf370511-bib-0123] Salee, N. , S. Naruenartwongsakul , W. Chaiyana , et al. 2023. “Comparison of Pulse Electric Field, Microwave and Ultrasonic Pretreatment Prior to Black Rice Extraction on Antioxidant and Sirtuin1 Enzyme Stimulating Activities.” Food Science and Technology 43: e123022. 10.1590/fst.123022.

[crf370511-bib-0124] Samsalee, N. , J. Meerasri , and R. Sothornvit . 2023. “Rice Husk Nanocellulose: Extraction by High‐Pressure Homogenization, Chemical Treatments and Characterization.” Carbohydrate Polymer Technologies and Applications 6: 100353. 10.1016/j.carpta.2023.100353.

[crf370511-bib-0125] San Sebastián, L. , E. P. Laveriano‐Santos , R. M. Lamuela‐Raventós , et al. 2026. “Green Optimisation of Microwave‐Assisted Extraction of Phenolic Compounds From Rice Straw Lignocellulosic Residues and LC‐HRMS‐Based Cultivar Profiling.” Food Chemistry 511: 148723. 10.1016/j.foodchem.2026.148723. 41825384

[crf370511-bib-0126] Sapwarobol, S. , W. Saphyakhajorn , and J. Astina . 2021. “Biological Functions and Activities of Rice Bran as a Functional Ingredient: A Review.” Nutrition and Metabolic Insights 14: 11786388211058559. 10.1177/11786388211058559. 34898989 PMC8655829

[crf370511-bib-0127] Sarfarazi, M. , S. M. Jafari , G. Rajabzadeh , and C. M. Galanakis . 2020. “Evaluation of Microwave‐Assisted Extraction Technology for Separation of Bioactive Components of Saffron (*Crocus sativus* L.).” Industrial Crops and Products 145: 111978.

[crf370511-bib-0128] Saveboworn, W. , S. Rittisak , R. Schoenlechner , S. Wangtueai , and R. Charoen . 2025. “Development of Functional Beverage With Rice Bran Protein (Hom Mali 105).” E3S Web of Conferences 610: 02004. 10.1051/e3sconf/202561002004.

[crf370511-bib-0129] Sengupta, R. , and D. K. Bhattacharyya . 1996. “Enzymatic Extraction of Mustard Seed and Rice Bran.” Journal of the American Oil Chemists' Society 73, no. 6: 687–692. 10.1007/BF02517941.

[crf370511-bib-0130] Sharma, A. , S. K. Khare , and M. N. Gupta . 2001. “Enzyme‐Assisted Aqueous Extraction of Rice Bran Oil.” Journal of the American Oil Chemists' Society 78, no. 9: 949–951. 10.1007/s11746-001-0369-x.

[crf370511-bib-0132] Sindhu, S. , S. Saloni , S. Sharma , and K. Chauhan . 2024. “Valorization of By‐Product From Rice Milling Industry by Enzymatic Hydrolysis and Potential Application.” Biomass Conversion and Biorefinery 14, no. 23: 29543–29559. 10.1007/s13399-023-05028-5.

[crf370511-bib-0133] Singh, R. , and M. Patel . 2022. “Effective Utilization of Rice Straw in Value‐Added By‐Products: A Systematic Review of State of Art and Future Perspectives.” Biomass and Bioenergy 159: 106411. 10.1016/j.biombioe.2022.106411.

[crf370511-bib-0134] Soares, J. F. , V. D. Prá , F. M. Barrales , et al. 2018. “Extraction of Rice Bran Oil Using Supercritical CO_2_ Combined With Ultrasound.” Brazilian Journal of Chemical Engineering 35, no. 2: 785–794. 10.1590/0104-6632.20180352s20160447.

[crf370511-bib-0175] Sowbhagya, H. B. , and V. N. Chitra . 2010. “Enzyme‐Assisted Extraction of Flavorings and Colorants from Plant Materials.” Critical Reviews in Food Science and Nutrition 50, no. 2: 146–161. 10.1080/10408390802248775. 20112157

[crf370511-bib-0135] Spaggiari, M. , C. Dall'Asta , G. Galaverna , and M. D. del Castillo Bilbao . 2021. “Rice Bran By‐Product: From Valorization Strategies to Nutritional Perspectives.” Foods 10, no. 1: 85. 10.3390/foods10010085. 33406743 PMC7824317

[crf370511-bib-0136] Steven, S. , E. Restiawaty , and Y. Bindar . 2021. “Routes for Energy and Bio‐Silica Production From Rice Husk: A Comprehensive Review and Emerging Prospect.” Renewable and Sustainable Energy Reviews 149: 111329. 10.1016/j.rser.2021.111329.

[crf370511-bib-0137] Tabaraki, R. , and A. Nateghi . 2011. “Optimization of Ultrasonic‐Assisted Extraction of Natural Antioxidants From Rice Bran Using Response Surface Methodology.” Ultrasonics Sonochemistry 18: 1279–1286. 10.1016/j.ultsonch.2011.05.004. 21612968

[crf370511-bib-0138] Talha, M. , S. Khalid , A. A. Maan , et al. 2024. “Ohmic Assisted Extraction: A Sustainable and Environment Friendly Approach to Substitute Conventional Extraction Methods.” Food Reviews International 40, no. 10: 3508–3529. 10.1080/87559129.2024.2366841.

[crf370511-bib-0139] Terigar, B. G. , S. Balasubramanian , C. M. Sabliov , M. Lima , and D. Boldor . 2011. “Soybean and Rice Bran Oil Extraction in a Continuous Microwave System: From Laboratory‐to Pilot‐Scale.” Journal of Food Engineering 104: 208–217. 10.1016/j.jfoodeng.2010.12.012.

[crf370511-bib-0140] Thongkong, S. , W. Klangpetch , K. Unban , et al. 2023. “Impacts of Electroextraction Using the Pulsed Electric Field on Properties of Rice Bran Protein.” Foods 12, no. 4: 835. 10.3390/foods12040835. 36832910 PMC9956254

[crf370511-bib-0141] Tomita, K. , S. Machmudah , R. Fukuzato , et al. 2014. “Extraction of Rice Bran Oil by Supercritical Carbon Dioxide and Solubility Consideration.” Separation and Purification Technology 125: 319–325. 10.1016/j.seppur.2014.02.008.

[crf370511-bib-0142] Totaro, G. , L. Sisti , M. Vannini , et al. 2018. “A New Route of Valorization of Rice Endosperm By‐Product: Production of Polymeric Biocomposites.” Composites Part B: Engineering 139: 195–202. 10.1016/j.compositesb.2017.11.055.

[crf370511-bib-0143] Trevisani Juchen, P. , M. Nolasco Araujo , F. Hamerski , M. L. Corazza , and F. A. Pedersen Voll . 2019. “Extraction of Parboiled Rice Bran Oil With Supercritical CO_2_ and Ethanol as Co‐Solvent: Kinetics and Characterization.” Industrial Crops and Products 139: 111506. 10.1016/j.indcrop.2019.111506.

[crf370511-bib-0144] Tufail, T. , H. B. U. Ain , J. Chen , et al. 2024. “Contemporary Views of the Extraction, Health Benefits, and Industrial Integration of Rice Bran Oil: A Prominent Ingredient for Holistic Human Health.” Foods 13, no. 9: 1305. 10.3390/foods13091305. 38731675 PMC11083700

[crf370511-bib-0145] Usman, M. , M. Nakagawa , and S. Cheng . 2023. “Emerging Trends in Green Extraction Techniques for Bioactive Natural Products.” Processes 11, no. 12: 3444. 10.3390/pr11123444.

[crf370511-bib-0146] Valles, A. , F. J. Álvarez‐Hornos , V. Martínez‐Soria , P. Marzal , and C. Gabaldón . 2020. “Comparison of Simultaneous Saccharification and Fermentation and Separate Hydrolysis and Fermentation Processes for Butanol Production From Rice Straw.” Fuel 282: 118831. 10.1016/j.fuel.2020.118831.

[crf370511-bib-0147] Van Hung, N. , M. C. Maguyon‐Detras , M. V. Migo , et al. 2020. “Rice Straw Overview: Availability, Properties, and Management Practices.” In Sustainable Rice Straw Management. Springer International Publishing. 10.1007/978-3-030-32373-8_1.

[crf370511-bib-0174] Wagner, H.‐E. , R. Brandenburg , K. V. Kozlov , A. Sonnenfeld , P. Michel , and J. F. Behnke . 2023. “The Barrier Discharge: Basic Properties and Applications to Surface Treatment.” Vacuum 71: 417–436. 10.1016/S0042-207X(02)00765-0.

[crf370511-bib-0148] Wang, C. H. , C. R. Chen , J. J. Wu , L. Y. Wang , C. M. J. Chang , and W. J. Ho . 2008. “Designing Supercritical Carbon Dioxide Extraction of Rice Bran Oil That Contain Oryzanols Using Response Surface Methodology.” Journal of Separation Science 31: 1399–1407. 10.1002/jssc.200700583. 18401861

[crf370511-bib-0149] Wang, N. , J. Chen , Q. Zhou , et al. 2021. “Crude Wax Extracted From Rice Bran Oil Improves Oleogel Properties and Oxidative Stability.” European Journal of Lipid Science and Technology 123, no. 6: 2000091. 10.1002/ejlt.202000091.

[crf370511-bib-0150] Wang, N. , X. Cui , Y. Duan , et al. 2023. “Potential Health Benefits and Food Applications of Rice Bran Protein: Research Advances and Challenges.” Food Reviews International 39, no. 6: 3578–3601. 10.1080/87559129.2021.2013253.

[crf370511-bib-0151] Wang, S. , T. Wang , Y. Sun , Y. Cui , G. Yu , and L. Jiang . 2022. “Effects of High Hydrostatic Pressure Pretreatment on the Functional and Structural Properties of Rice Bran Protein Hydrolysates.” Foods 11, no. 1: 29. 10.3390/foods11010029. PMC8749986 35010157

[crf370511-bib-0152] Wang, Z. , S. Li , M. Zhang , et al. 2022. “Optimization of Oil Extraction From Rice Bran With Mixed Solvent Using Response Surface Methodology.” Foods 11: 3849. 10.3390/foods11233849. 36496655 PMC9737536

[crf370511-bib-0153] Wei, M. C. , J. Xiao , and Y. C. Yang . 2016. “Extraction of Alpha‐Humulene‐Enriched Oil From Clove Using Ultrasound‐Assisted Supercritical Carbon Dioxide Extraction and Studies of Its Fictitious Solubility.” Food Chemistry 210: 172–181. 10.1016/j.foodchem.20. 27211636

[crf370511-bib-0154] Wongthaweewatana, I. , T. R. Srinophakun , I. Saramala , and K. Kasemwong . 2021. “Production of Milk Analogues From Rice Bran Protein Hydrolysate Using the Subcritical Water Technique.” Food Science and Technology 41, no. 3: 722–729. 10.1590/fst.16520.

[crf370511-bib-0155] Wu, C. , Y. Zhang , J. Wang , W. Xu , Z. Hu , and C. Hu . 2023. “Continuous Enzyme Crosslinking Modifying Colloidal Particle Characteristics and Interface Properties of Rice Bran Protein to Improve the Foaming Properties.” LWT 184: 114997. 10.1016/j.lwt.2023.114997.

[crf370511-bib-0156] Xi, J. , Y. Wang , X. Zhou , S. Wei , and D. Zhang . 2024. “Cold Plasma Pretreatment Technology for Enhancing the Extraction of Bioactive Ingredients From Plant Materials: A Review.” Industrial Crops and Products 209: 117963. 10.1016/j.indcrop.2023.117963.

[crf370511-bib-0157] Xu, D. , J. Hao , Z. Wang , et al. 2021. “Physicochemical Properties, Fatty Acid Compositions, Bioactive Compounds, Antioxidant Activity and Thermal Behavior of Rice Bran Oil Obtained With Aqueous Enzymatic Extraction.” LWT 149: 111817. 10.1016/j.lwt.2021.111817.

[crf370511-bib-0158] Yadav, S. , S. Mishra , and R. C. J. U. S. Pradhan . 2021. “Ultrasound‐Assisted Hydration of Finger Millet (*Eleusine coracana*) and Its Effects on Starch Isolates and Antinutrients.” Journal of Pharmaceutical Sciences 73: 105542. 10.1016/j.ultsonch.2021.105542. PMC8050032 33819868

[crf370511-bib-0159] Yeboah, W. O. , E. M. Kwofie , and D. Wang . 2022. “Circular Bioeconomy Potential of Rice Husk as a Bioplastic Resource: Techno‐Environmental Assessment.” Bioresource Technology Reports 20: 101248. 10.1016/j.biteb.2022.101248.

[crf370511-bib-0160] Yılmaz Tuncel, N. 2023. “Stabilization of Rice Bran: A Review.” Foods 12, no. 9: 1924. 10.3390/foods12091924. 37174460 PMC10178138

[crf370511-bib-0161] Yuan, S. , Y. Hou , S. Liu , and Y. Ma . 2024. “A Comparative Study on Rice Husk, as Agricultural Waste, in the Production of Silica Nanoparticles via Different Methods.” Materials 17, no. 6: 1271. 10.3390/ma17061271. 38541425 PMC10972294

[crf370511-bib-0162] Zadeike, D. , R. Vaitkeviciene , R. Degutyte , et al. 2022. “A Comparative Study on the Structural and Functional Properties of Water‐Soluble and Alkali‐Soluble Dietary Fibres From Rice Bran After Hot‐Water, Ultrasound, Hydrolysis by Cellulase, and Combined Pre‐Treatments.” International Journal of Food Science & Technology 57, no. 2: 1137–1149. 10.1111/ijfs.15480.

[crf370511-bib-0163] Zaky, A. A. , A. M. Abd El‐Aty , A. Ma , and Y. Jia . 2022. “An Overview on Antioxidant Peptides From Rice Bran Proteins: Extraction, Identification, and Applications.” Critical Reviews in Food Science and Nutrition 62, no. 5: 1350–1362. 10.1080/10408398.2020.1842324. 33146021

[crf370511-bib-0164] Zeng, Z. , Y. Wang , G. Xu , L. Zhou , C. Liu , and S. Luo . 2023. “Peroxidase Inactivation by Cold Plasma and Its Effects on the Storage, Physicochemical and Bioactive Properties of Brown Rice.” Food Bioscience 52: 102383. 10.1016/j.fbio.2023.102383.

[crf370511-bib-0165] Zhang, S. , Z. Zhang , M. Wang , J. Jia , and Q. Wu . 2024. “Effects of Rice Bran Soluble Dietary Fiber on the Physicochemical Properties of Frozen Dough and the Quality of Steamed Bread.” LWT 214: 117120. 10.1016/j.lwt.2024.117120.

[crf370511-bib-0166] Zhang, W. , X. Xu , Y. Yuan , and Z. Wang . 2023. “Sustainable Application of Rice‐Waste for Fuels and Valuable Chemicals—A Mini Review.” Frontiers in Chemistry 11: 1225073. 10.3389/fchem.2023.1225073. 37927567 PMC10620727

[crf370511-bib-0167] Zhao, M. , Y. Lan , L. Cui , E. Monono , J. Rao , and B. Chen . 2020. “Formation, Characterization, and Potential Food Application of Rice Bran Wax Oleogels: Expeller‐Pressed Corn Germ Oil Versus Refined Corn Oil.” Food Chemistry 309: 125704. 10.1016/j.foodchem.2019.125704. 31699556

[crf370511-bib-0168] Zheng, S. , F. Wei , Q. Zheng , J. Xiao , Y. Cao , and Y. Lan . 2023. “Fabrication of Rice Bran Protein—Sodium Alginate—Calcium Double Cross‐Linked Foam Template for Oleogel Preparation.” Food Hydrocolloids 143: 108873. 10.1016/j.foodhyd.2023.108873.

[crf370511-bib-0169] Zhou, L. , J. Huang , Y. Du , et al. 2025. “Non‐Thermal Stabilization Strategies for Rice Bran: Mechanistic Insights, Technological Advances, and Implications for Industrial Applications.” Foods 14, no. 9: 1448. 10.3390/foods14091448. 40361531 PMC12071984

[crf370511-bib-0170] Zhuang, X. , T. Yin , W. Han , and X. Zhang . 2019. “Nutritional Ingredients and Active Compositions of Defatted Rice Bran.” In Rice Bran and Rice Bran Oil, edited by L.‐Z. Cheong and X. Xu , 247–270. AOCS Press. 10.1016/B978-0-12-812828-2.00010-X.

[crf370511-bib-0171] Zullaikah, S. , Y. A. Pujiastuti , G. Prihandini , and M. Rachimoellah . 2019. “Wet Extraction of γ‐Oryzanol From Rice Bran.” IOP Conference Series: Materials Science and Engineering 543, no. 1: 012078. 10.1088/1757-899X/543/1/012078.

